# Antibiotic stress-induced modulation of the endoribonucleolytic activity of RNase III and RNase G confers resistance to aminoglycoside antibiotics in *Escherichia coli*

**DOI:** 10.1093/nar/gku093

**Published:** 2014-01-30

**Authors:** Wooseok Song, Yong-Hak Kim, Se-Hoon Sim, Soonhye Hwang, Jung-Hyun Lee, Younghoon Lee, Jeehyeon Bae, Jihwan Hwang, Kangseok Lee

**Affiliations:** ^1^Department of Life Science, Chung-Ang University, Seoul 156-756, Republic of Korea, ^2^Marine Biotechnology Research Division, Korea Institute of Ocean Science and Technology, Ansan 426-744, Republic of Korea, ^3^Department of Microbiology, Catholic University of Daegu, School of Medicine, Nam-Gu, Daegu 705-718, Republic of Korea, ^4^Department of Chemistry, KAIST, Daejeon 305-701, Republic of Korea, ^5^Department of Pharmacy, Chung-Ang University, Seoul 156-756, Republic of Korea and ^6^Department of Microbiology, Pusan National University, Busan 609-735, Republic of Korea

## Abstract

Here, we report a resistance mechanism that is induced through the modulation of 16S ribosomal RNA (rRNA) processing on the exposure of *Escherichia coli* cells to aminoglycoside antibiotics. We observed decreased expression levels of RNase G associated with increased RNase III activity on *rng* mRNA in a subgroup of *E. coli* isolates that transiently acquired resistance to low levels of kanamycin or streptomycin. Analyses of 16S rRNA from the aminoglycoside-resistant *E. coli* cells, in addition to mutagenesis studies, demonstrated that the accumulation of 16S rRNA precursors containing 3–8 extra nucleotides at the 5’ terminus, which results from incomplete processing by RNase G, is responsible for the observed aminoglycoside resistance. Chemical protection, mass spectrometry analysis and cell-free translation assays revealed that the ribosomes from *rng*-deleted *E. coli* have decreased binding capacity for, and diminished sensitivity to, streptomycin and neomycin, compared with wild-type cells. It was observed that the deletion of *rng* had similar effects in *Salmonella enterica* serovar Typhimurium strain SL1344. Our findings suggest that modulation of the endoribonucleolytic activity of RNase III and RNase G constitutes a previously uncharacterized regulatory pathway for adaptive resistance in *E. coli* and related gram-negative bacteria to aminoglycoside antibiotics.

## INTRODUCTION

Approximately 50% of naturally produced antibiotics target the bacterial ribosome by binding to one of three principal ribosomal sites: the decoding site, the peptidyl transferase center and the peptide exit tunnel. Among these antibiotics, macrolides, aminoglycosides, tetracyclines, glycyclines and their derivatives are well known to inhibit protein synthesis by binding to distinct sites on bacterial ribosomes (1–3). However, the clinical use of these and many other antibiotics has been compromised by the emergence of resistant bacteria. The mechanisms of resistance predominantly involve (i) enzymatic modifications of the antibiotics and their ribosomal target sites and (ii) the operation of drug efflux pumps. In addition, some gram-negative bacteria with noninherited resistance to a broad range of antibiotics are able to cause persistent infections (4,5). However, the precise mechanism for the causal relationship between bacterial persistence and antibiotic resistance has not been clearly identified.

The detailed mechanism by which antibiotics interact with ribosomes has long been investigated (6,7). In addition to biochemical and genetic analyses of ribosomes, recent studies of the three-dimensional structure of antibiotics bound to bacterial ribosomes using cryo-electron microscopy have clearly demonstrated that ribosomal RNAs (rRNAs) play pivotal roles in the interaction between antibiotics and ribosomes (1,2). The structural motifs for antibiotic binding are mostly composed of rRNAs, and alterations in these rRNAs, including base modification or nucleotide substitution, influence the susceptibility of ribosomes to antibiotics. A recent report revealed that the presence of an extra 66 nucleotides (nt) at the 5′ terminus of the 16S rRNA, which results from nucleotide substitution or incomplete processing of the 16S rRNA, can also lead to an increased sensitivity of *Escherichia coli* cells to certain aminoglycoside antibiotics (8).

In *E. coli*, as in most other organisms, the rRNA genes (rDNA) encode rRNAs in a single transcriptional unit that undergoes extensive processing to generate fully mature rRNAs. The endoribonuclease RNase III (encoded by *rnc*) first cleaves the 30S primary transcripts to yield the 17S, p23S and 9S rRNA precursors for 16S, 23S and 5S rRNAs, respectively (9–11). The 17S rRNA contains an additional 115 and 33 nt at the 5′ and 3′ ends, respectively. Maturation of the 3′ end of the 17S rRNA is catalyzed by four 3′ to 5′ exoribonucleases: RNase II, RNase R, RNase PH and polynucleotide phosphorylase (PNPase), and recently characterized YbeY (12,13). In contrast, maturation of the 5′ end of the 17S rRNA occurs via the sequential action of the endoribonucleases RNase E and RNase G, which generate the 16.3S precursor containing 66 extra nt at the 5′-end and mature 16S rRNA, respectively (14,15). The individual inactivation of these ribonucleases results in the accumulation of rRNA precursors, which generally undergo complete processing and are assembled into 70S ribosomes, indicating the presence of alternative rRNA processing pathways (10,14,15). The exception to this phenomenon involves the rRNA precursors that accumulate in *E. coli* cells deleted for the gene encoding RNase G (*rng*). Although the ribosomes in these cells contain incompletely processed 16S rRNA, they appear to be as efficient as wild-type ribosomes with respect to performing protein synthesis (16) and supporting the normal growth of *E. coli* (17).

While performing experiments aimed at characterizing changes in the proteome profiles of wild-type *E. coli* cells that had transiently acquired ‘noninherited resistance’ to aminoglycoside antibiotics, we observed that some of the resistant cells had significantly decreased cellular levels of RNase G. This observation led us to investigate whether the downregulation of RNase G is sufficient to cause the accumulation of incomplete 16S rRNA, thereby affecting the susceptibility of the ribosome to aminoglycoside antibiotics. In this study, we performed biochemical and genetic analyses to examine 16S rRNA processing by RNase G in two *E. coli* K-12 strains (MG1655 and N3433) and *Salmonella enterica* serovar Typhimurium strain SL1334. Our experimental results show that the presence of 3–8 extra nt at the 5′ terminus of the 16S rRNA, which results from incomplete processing by RNase G, is sufficient to induce aminoglycoside resistance. We further show that ribosomes containing these incompletely processed 16S rRNAs have decreased binding capacity and susceptibility to aminoglycoside antibiotics. In addition, we demonstrate that decreased RNase G expression in aminoglycoside-resistant *E. coli* isolates is likely associated with increased RNase III cleavage activity on *rng* mRNA by an as-yet-unidentified mechanism that is initiated on the exposure of the cells to aminoglycosides. Our findings suggest that certain degrees of resistance to aminoglycoside antibiotics in *E. coli* and related gram-negative bacteria are attributable to this RNase III- and RNase G-mediated regulatory pathway.

## MATERIALS AND METHODS

### Bacterial strains

All experiments were performed with the *E. coli* strains N3433 (*Hfr, lacZ43, λ-, relA1, spoT1, thi-1*) and MG1655 (*F^−^, lambda^−^, rph-1*) and *S. enterica* SL1344 and their derivatives. N3433*rng*, MG1655*rng* and *S. enterica* SL1344*rng* (*rng*-deletion strains) were constructed by the one-step inactivation of the chromosomal gene using the method described by Datsenko and Wanner (18). To construct N3433*rng* and MG1655*rng*, the primers 5′-*rng*-KO (5′-GTGAGAAAAGGGATAAACATGACGGCTGAATTGTTAGTAAACGTAACGGTGTAGGCTGGAGCTGCTTC-3′, the complementary sequence of 16 codons of the 5′-*rng* ORF are underlined) and 3′-*rng*-KO (5′-TTACATCATTACGACGTCAAACTGCTCCTGGTTATAGAGCGGTTCAATATTCCGGGGATCCGTCGACC-3′, the complementary sequence of 16 codons of the 3′-*rng* ORF are underlined) were used to amplify the kanamycin antibiotic resistance marker in pKD13 (18). To construct *S. enterica* SL1344*rng*, the primers 5′-*rng*-P1 (5′-ATGACGGCTGAATTGTTGGTAAACGTAACGCCATCGGAAGTGTAGGCTGGAGCTGCTTC-3′, the complementary sequence of 13 codons of the 5′-*rng* ORF are underlined) and 3′-*rng*-P2 (5′-ATCCGGCCTGCAAATACTCGTACGGGTCGCGGTCTTTTACATATGAATATCCTCCTTA-3′, the complementary sequence of 40 nts of the 3′-*rng* ORF are underlined) were used to amplify the chloramphenicol antibiotic resistance marker in pKD3 (18). Replacement of the resistance marker with the *rng* gene and subsequent removal of the antibiotic markers were confirmed by PCR amplification of the chromosomal region encompassing the *rng* locus using the primers *rng*-5′-UTR (5′-GTGGATTTTCTTGCTGATGC-3′) and *rng*-3′ (5′-ATTACGACGTCAAACTGCTC-3′) for *E. coli* and *rng*-F-con (5′-CGCACTGCGTGATAAAAGGG-3′) and *rng*-R-con (5′-GAACGCCTTATCCGGCCTGC-3′) for *S. enterica* SL1344. The construction of KSL2000 (*rne-*deletion strain) and the depletion of RNase E in KSL2000 have been previously described (19). The *E. coli* strain KSC004 (20) was obtained from Dr. Stanley N. Cohen.

To introduce three nucleotides in the RNase G cleavage site at the 5′ end of the 16S rRNA in pRNA9, DNA fragments containing a 3-nt insertion in the RNase G cleavage site at the 5′-end of the 16S rRNA were amplified using the overlap extension PCR method (21) with the following primers: 16S-ins3N-F (5′-ATTACGAAGTTTAATTCTTTGAGCGTCAAACTTTTNNNAAATTGAAGAGTTTGATCATGGCTCA-3′), 16S-5R (5′-GTTTGACGCTCAAAGAATTAAACTTCGTA-3′), 16S-418F (5′-AGCTGTTGCCCGTCTCACTG-3′) and 16S-812R (5′-CGGCGTGGACTACCAGGGTA-3′).

The plasmid pRNC3, which contains a cloned copy of the *rnc* gene, was constructed by subcloning a NotI-XbaI fragment containing the coding region of RNase III from pRNC1 into the same site in pPM30 (22).

### Isolation of *E. coli* cells with naturally induced resistance to aminoglycosides

Cells were grown in LB medium at 37°C to an optical density at 600 nm (OD_600_) of 0.6. A 50-μl aliquot of cells was spread onto LB agar plates with or without the addition of an aminoglycoside. Kanamycin (10 μg/ml), neomycin (5 μg/ml) or streptomycin (10 μg/ml) was used for the isolation of *E. coli* cells that had acquired a naturally induced resistance to aminoglycosides. After a 24-h incubation at 37°C, 1 × 10^4^ colonies were formed on LB agar plates without aminoglycoside, whereas 20–30 colonies were formed on the plates containing each of the aminoglycosides.

### Western blot analysis

The western blot analyses were performed as described previously (23). Polyclonal antibodies against RNase III were obtained from rabbits injected with purified His-tagged RNase III, as described (24). Polyclonal antibodies against RNase G and a monoclonal antibody against RNase E were obtained from Dr. Stanley N. Cohen. Images of the western blots were obtained using a VersaDoc 100 system (Bio-Rad), and the densities of each band were quantified with Quantity One software (Bio-Rad). Ribosomal protein S1 was used as a control. The relative abundance of the protein bands was quantified by setting the amount of RNase G or RNase III protein produced in wild-type cells to one.

### Total RNA isolation and primer extension analysis

Total cellular RNAs were extracted from cells at OD_600_ = 0.6 using an RNeasy mini prep kit (Qiagen). rRNAs were purified from ribosomes using phenol–chloroform extraction followed by ethanol precipitation. Primers were labeled at the 5′-end using [γ-^32^P]ATP and T4 polynucleotide kinase. RNA and labeled primers were hybridized at 65°C for 5 min, and the reaction was then cooled to 37°C. The extension reaction was performed with Avian Myeloblastosis Virus (AMV) reverse transcriptase at 42°C for 1 h, and the extended fragments were analyzed on a 10% polyacrylamide gel containing 8 M urea. The 16S rRNA-rng primer (5′-CAGCGTTCAATCTGAGCCATGATC-3′) was used for the analysis of the 5′-end processing of the 16S rRNA. A sequencing reaction was performed and used as a molecular weight marker.

### Preparation of ribosomes and *E. coli* S30 extract

The 30S subunits and 70S ribosomes were isolated as described previously (25,26). The S30 extracts used for the cell-free translation system were prepared from *E. coli* strains N3433 and N3433*rng*, as described previously (27).

### Cell-free translation assays

*In vitro* translation reactions were performed as described previously with minor modifications (27). The S30 extract was incubated at 37°C for 90 min in a premix [87.5 mM Tris-acetate (pH 8.0), 476 mM potassium glutamate, 75 mM CH_3_COO NH_4_, 5 mM DTT, 20 mM Mg[CH_3_COOH]_2_, 1.25 mM each of 19 amino acids (except for methionine), 5 mM ATP, 1.25 mM GTP, 50 mM phosphoenolpyruvate, 2.5 mg/ml *E. coli* tRNAs, 6.25% glycerol and 50 µg/ml folinic acid] containing [^35^S]-methionine, synthetic CAT or lacZα mRNA and antibiotics at increasing concentrations. The *in vitro* synthesis of CAT or lacZα mRNA was performed as described previously by Lee and Cohen (28). The protein products were analyzed by 12% sodium dodecyl sulfate–polyacrylamide gel electrophoresis (SDS-PAGE) for CAT or 13% Tricine-SDS-PAGE for LacZα.

### Binding of streptomycin and neomycin to 30S subunits

The binding of streptomycin and neomycin to the 30S subunits was performed as described by Spickler *et al.* (29), with minor modifications. Heat activation of 30S subunits (50 pmol) was performed at 37°C for 20 min in binding buffer [70 mM HEPES (pH 7.8), 20 mM MgCl_2_, 300 mM KCl, 0.5 mM EDTA and 2 mM DTT]. Streptomycin and neomycin were then added, and the mixture was first incubated at 37°C for 30 min and subsequently maintained on ice for 30 min.

### Probing with DMS

The protocol for probing with dimethyl sulfate (DMS) was adapted from standard procedures (29–31). DMS (1 μl) was added to 200 µl of binding buffer (see above) containing 50 pmol of 30S subunits purified from N3433 and N3433*rng* cells. The DMS reaction was performed for 10 min at 37°C in the presence or absence of streptomycin and neomycin. The modification sites were determined by primer extension; the primers used to detect the 3′ minor domain were 16S-1506R (5′-CAGGTTCCCCTACGGTTA-3′) and 16S-1424R (5′-TACCTACTTCTTTTGCAA-3′).

### β-Galactosidase assay

The β-galactosidase activity in whole cells was determined as described previously (32).

### Mass spectrometry

To analyze a difference in molecular ratio of streptomycin bound to 30S subunits extracted from N3433 and N3433*rng* strains, each 15 pmol 30S dose was treated with 0, 0.03, 3 or 300 nmol dose of streptomycin in the binding buffer, as described above, and unbound streptomycin and salts were removed using protein desalting spin columns (illusta™ MicroSpin™ G-25 Columns, GE Healthcare). The 30S eluant was treated with 0.2% trifluoroacetic acid to dissociate streptomycin on ice, and was spiked with 1.5 nmol dose of amikacin, gentamicin or kanamycin, which were used as internal standard for quantification of streptomycin by mass spectrometry. After centrifugation at 4°C and 40 000rpm using a Beckman 70 Ti rotor, 2 μl supernatant was injected to a Thermo Velos Pro Mass instrument equipped with an Accela 600 pump system, which delivered 0.1% formic acid at a flow rate of 100 μl/min. The full mass scan in a range of *m/z* 150 to 600 was performed in positive ion mode at capillary voltage of 4 keV, and followed by tandem mass spectrometry for monoisotopic mass ion [MH]^+^ peaks of amikacin (*m/z* = 586.29), gentamicin (*m/z* = 478.32), kanamycin (*m/z* = 485.25) and streptomycin (*m/z* = 582.27). For quantification, standard curves were constructed by mixing 1.5 nmol each internal standard with various concentrations of streptomycin in a range of 0.015 to 15 nmol in 30S subunits treated with 0.2% trifluoroacetic acid. In this range, relative signal intensities of streptomycin to amikacin showed the best linearity (*r*^2^ > 0.999) with the limit of detection at 2.36 pmol and the limit of quantification at 7.87 pmol, so this standard curve was used for calculation of molecular number of streptomycin in each sample. The calculated number of streptomycin was divided by that of 30S subunit eluted from desalting column to determine the ratio of streptomycin bound to 30S subunit. The molecular number of 30S subunit was estimated by relative band intensities of 30S subunit proteins in a SDS-PAGE gel stained with Coomassie Brilliant R-250, before and after being applied onto the protein desalting columns.

### Statistical analysis

Experimental data that were obtained from at least three independent experiments are expressed using means and standard errors. Statistical analysis was performed by one-way analysis of variance (ANOVA) and t-test using IBM SPSS Statistics 2.0. Statistically significant values are marked with asterisks (*P < 0.1, **P < 0.05, ***P < 0.01).

## RESULTS

### Noninherited aminoglycoside resistance is related to RNase G expression in *E. coli*

**Figure 1.**
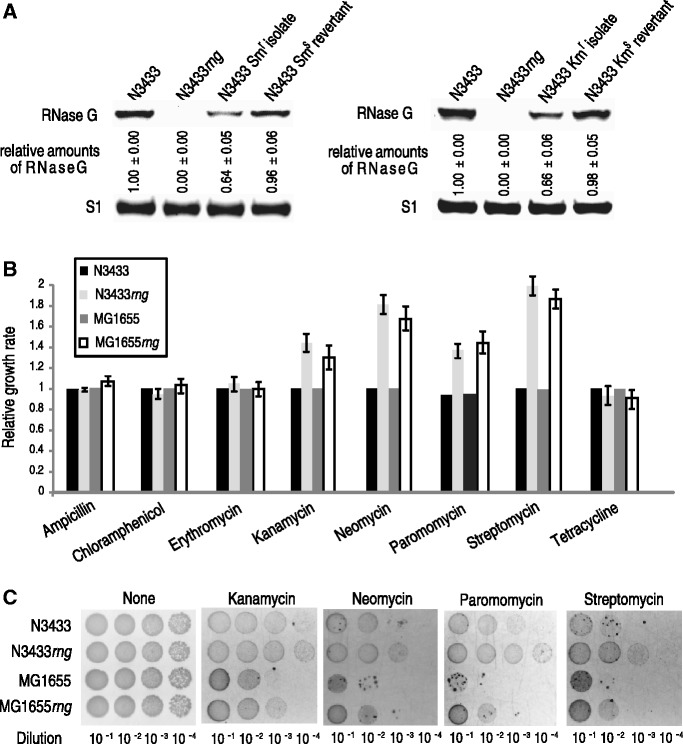
Relationship between RNase G expression levels and aminoglycoside resistance in *E. coli*. (**A**) Correlation between RNase G expression levels and noninherited resistance to aminoglycosides. The cellular levels of RNase G in naturally induced streptomycin-resistant (Sm^r^) or kanamycin-resistant (Km^r^) isolates and their revertants (Sm^s^ or Km^s^) were estimated by a western blot analysis. Sm^r^ and Km^r^ isolates were grown in LB medium containing 3 μg/ml of streptomycin or kanamycin; the Sm^r^ and Km^r^ isolates were then cultured in LB medium without the addition of antibiotics to restore the aminoglycoside sensitivity of the wild-type N3433 strain. Total proteins were prepared from cultures with an OD at 600 nm (OD_600_) of approximately 0.6. (**B**) Effects of the *rng* deletion on the sensitivity of *E. coli* N3433 and MG1655 to antibiotics. The cells were grown to mid-log phase at 37°C and diluted 1:100 with LB containing different concentrations of antibiotics (6 μg/ml ampicillin, 2 μg/ml chloramphenicol, 50 μg/ml erythromycin, 3 μg/ml kanamycin, 1 μg/ml neomycin, 5 μg/ml paromomycin, 3 μg/ml streptomycin or 1 μg/ml tetracycline), followed by further incubation for 12 h at 37°C. After the 12-h incubation, the cell growth was measured at OD_600_. (**C**) Effect of the *rng* deletion on the sensitivity of the N3433 and MG1655 strains to aminoglycosides. The cells were grown to mid-log phase (OD_600_ = 0.6) at 37°C. The cells were then diluted 10*^−^*^1^–10*^−^*^4^ and spotted onto LB agar plates containing kanamycin (3 μg/ml), neomycin (1 μg/ml), paromomycin (5 μg/ml) or streptomycin (3 μg/ml). The plates were incubated for 12 h at 37°C and analyzed.

We isolated aminoglycoside-resistant *E. coli* colonies to identify factors conferring resistance to low levels of aminoglycoside antibiotics. Of 1 × 10^4^ *E. coli* N3433 cells, 20–30 cells formed colonies on selective Luria-Bertani (LB) media containing kanamycin or streptomycin (see ‘Materials and Methods’ for details regarding the procedure used for selecting resistant isolates). When analyzing the proteome profiles (Kim, Lee, Kwon, Yu and Lee, in preparation), we observed that aminoglycoside resistance is most strongly associated with decreased expression levels of RNase G and altered, albeit less significantly, expression levels of ribosomal subunits and associated proteins, presumably reflecting modifications of the antibiotic targets, in a subgroup of the isolates (Figure 1A). Additionally, we observed that the cells showing naturally induced resistance to these antibiotics lost the resistance phenotype and that the normal cellular levels of RNase G were restored when the cells were grown in a medium without antibiotics (Figure 1A). These results suggest that certain regulatory pathways that cause noninherited resistance to aminoglycosides in *E. coli* cells are related to RNase G expression.

### Deletion of *rng* promotes bacterial growth and resistance under aminoglycoside antibiotic stress

Because our results indicated a possible correlation between RNase G expression levels and aminoglycoside resistance in *E. coli*, we tested the susceptibility of stable *E. coli* strains that do not express RNase G to several different classes of antibiotics. The *E. coli* N3433 strain deleted for the *rng* gene (N3433*rng*) showed a marked increase in resistance to aminoglycoside antibiotics that target the ribosomal decoding site (kanamycin, neomycin, paromomycin and streptomycin) compared with its parental strain N3433 (Figure 1B and C). Both the wild-type and *rng*-deletion strains appeared to have a similar susceptibility to other antibiotics targeting either the bacterial cell wall (ampicillin) or ribosomal sites other than the decoding site (chloramphenicol, erythromycin and tetracycline). We also obtained analogous results using the MG1655 and MG1655*rng* strains, suggesting that the reduced susceptibility of *rng*-deleted *E. coli* cells to aminoglycoside antibiotics was not strain specific (Figure 1B and C). To further explore this difference, growth curves were measured in LB media in the presence or absence of antibiotics. As shown in Supplementary Figure S1, the N3433*rng* strain began growing at approximately 5 h in LB containing low levels of kanamycin, neomycin, paromomycin or streptomycin, whereas the wild-type strain began growing at 8 h under the same conditions. This suggests that the N3433*rng* strain adapted more rapidly to the aminoglycoside stress conditions compared with N3433. N3433 and N3433*rng* showed similar growth curves when cultured in nonselective LB media (Supplementary Figure S1). These results indicate that the *rng* deletion promotes the growth of *E. coli* cells to improve survival under antibiotic stress.

### Accumulation of 16S rRNA precursors containing an extra 3–8 nt is sufficient to confer aminoglycoside resistance in *E. coli*

**Figure 2.**
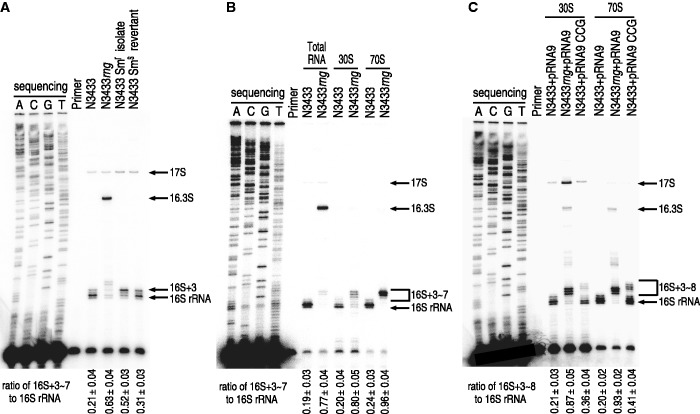
Analysis of the 5' end of 16S rRNA in aminoglycoside-resistant *E. coli*. (**A**) The 5'-end processing of 16S rRNA in the naturally induced streptomycin-resistant isolate and its revertant (used in Figure 1A) were analyzed by primer extension analysis. (**B**) Analysis of the 5' end of 16S rRNA in N3433*rng*. Total RNA, 30S subunits and 70S ribosomes were purified from N3433 and N3433*rng* cells and used for primer extension analysis. (**C**) Effects of CCG insertion on the 5'-end processing of 16S rRNA. A primer extension analysis was used to analyze the 30S subunits and 70S ribosomes prepared from strains N3433+pRNA9, N3433*rng*+pRNA9 and N3433+pRNA9CCG. Cultures were grown to early log phase (OD_600_ = 0.1), and 1 mM IPTG was added to induce the transcription of *rrn*B from a plasmid-borne promoter. The cultures were further grown to mid-log phase (OD_600_ = 0.6–0.7) at 37°C and harvested for the isolation of ribosomes.

To understand the molecular mechanisms of aminoglycoside resistance in *E. coli* cells that either do not express RNase G or express low levels of the enzyme, a primer extension analysis of 16S rRNA was performed using *rng* deletion mutants and aminoglycoside-resistant isolates. When total RNA extracts were examined, a streptomycin-resistant N3433 isolate exhibited an increased level of an incomplete 16S rRNA containing extra nucleotides at the 5′ end (designated as 16S+3); this rRNA was up to 2.5 times more abundant in this isolate compared with its levels in the original and revertant strains grown in nonselective media (Figure 2A). These results indicate a correlation between aminoglycoside resistance and the accumulation of 16S+3. The 16S+3 that accumulated in these isolates may reflect a lower 16S rRNA processing activity through decreased expression of RNase G. The accumulation of 16S+3 was also noted in N3433*rng* among minor 16S precursors that contained 3–7 extra nt at the 5′ end (designated as 16S+3∼7). However, the majority of the 16S rRNA precursors were 16.3S, containing 66 extra nt at the 5′-end. Although 16.3S accounted for the majority (87%) of the total 16S rRNA precursors, the primer extension analyses of the 5′ terminus of 16S rRNA from 70S ribosomes in N3433*rng* revealed that 96% of the 16S rRNA precursors were 16S+3∼7 (Figure 2B). The proportion of 16.3S rRNA was significantly decreased from 87% to 2% of the total 16S rRNA precursors in the N3433*rng* 70S ribosomes, indicating that the phenotype of aminoglycoside resistance may result from the accumulation of 16S+3∼7 rather than 16.3S. Furthermore, we observed that the effects of *rng* deletion on the accumulation of 16S+3∼7 in 70S ribosomes in the MG1655 strain were analogous to those described above (Supplementary Figure S2).

Given the above findings, we investigated the possibility that the accumulation of 16S+3∼7 is sufficient to account for the aminoglycoside resistance of *E. coli*. We inserted three random nucleotides upstream of the RNase G processing site at the 5′ terminus of the 16S rRNA gene in the rRNA overexpression plasmid pRNA9 (Supplementary Figure S3A) (33). The pRNA9 plasmid contains a cloned copy of *rrnB* that produces *E. coli* rRNA transcripts under the control of the isopropyl-thiogalactoside (IPTG)-inducible *lacUV5* promoter. Plasmid-derived rRNA accounted for 35–40% of the total rRNA in the cells carrying pRNA9 that were induced to express rRNA via the addition of 1 mM IPTG to the culture medium (33,34). We then screened a plasmid population mutagenized at the RNase G processing site of the 16S rRNA to identify streptomycin-resistant clones, which were defined as those that grew better than N3433 harboring pRNA9 on LB medium containing 2 μg/ml streptomycin in the presence of 1 mM IPTG. From 200 transformants tested, we obtained seven clones that exhibited better growth compared with N3433 harboring pRNA9 when plasmid DNA purified from these clones was retransformed into N3433 (Supplementary Figure S3A). Sequence analysis revealed that these clones had different triple-base insertions (Supplementary Figure S3A). Total RNAs were isolated from these clones for the analysis of the 5′ terminus of the 16S rRNA using primer extension, and an accumulation of 16S rRNAs containing 3–8 extra nt (designated as 16S+3∼8) was observed in these clones (Supplementary Figure S3B). One of these clones (pRNA9-CCG) was further analyzed for the accumulation of 16S+3∼8 in 70S ribosomes (Figure 2C). The results showed that 16S+3∼8 accounted for approximately 41% of the 16S rRNA species in the 70S ribosomes from N3433 harboring pRNA9-CCG, whereas 16S+3 accounted for ∼20% of that from N3433 harboring pRNA9. Approximately 93% of the 16S rRNA species in the 70S ribosomes from N3433*rng* harboring pRNA9 were 16S+3∼7. The extent of accumulation of 16S rRNAs containing 3–8 extra nt was correlated with the degree of aminoglycoside resistance: N3433*rng* harboring pRNA9 showed a stronger resistance to streptomycin than N3433 harboring pRNA9-CCG (Supplementary Figure S3C). N3433 harboring pRNA9-CCG also showed increased resistance to other aminoglycosides, such as kanamycin, neomycin and paromomycin (Supplementary Figure S3C). These results support our hypothesis that the accumulation of incomplete 16S rRNAs (e.g., 16S+3∼7) in the ribosome is sufficient to render *E. coli* cells resistant to aminoglycoside antibiotics.

### Effects of 16S+3∼7 accumulation on protein synthesis in the presence of aminoglycosides

**Figure 3.**
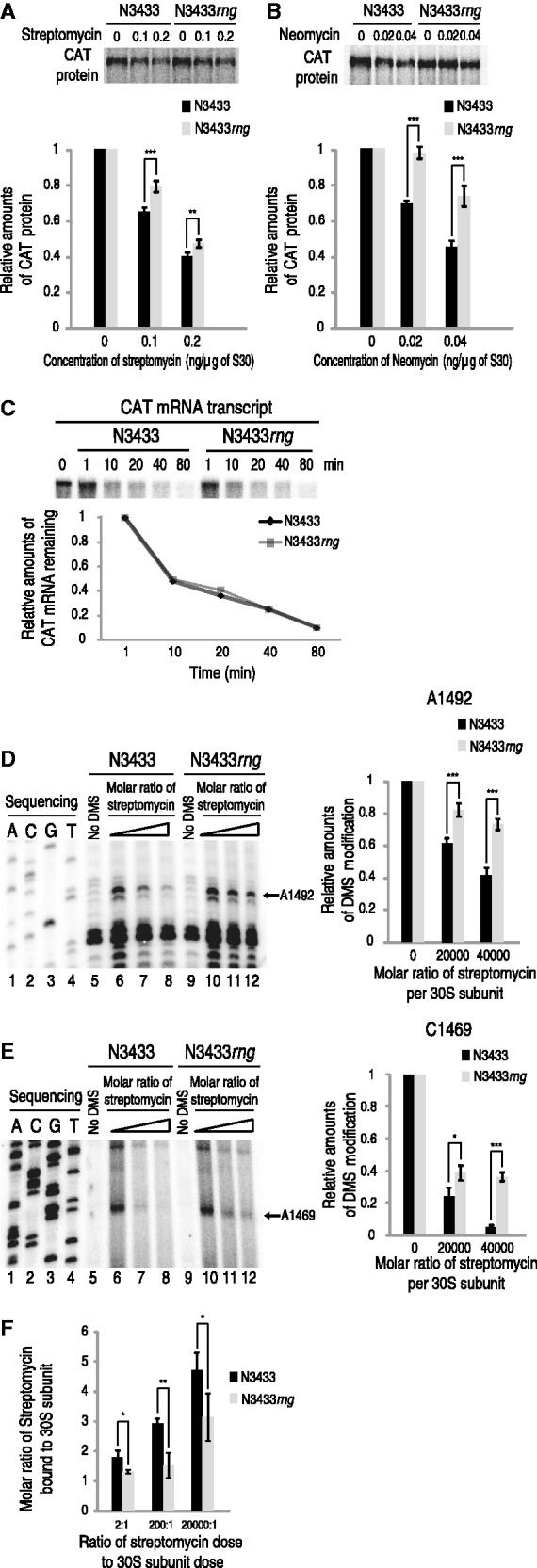
Effects of 16S+3∼7 accumulation on the sensitivity and binding capacity of ribosomes to aminoglycosides. (**A** and **B**) Effects of 16S+3∼7 accumulation on the sensitivity of ribosomes to aminoglycosides. *In vitro* translation was performed for CAT protein production using *in vitro-*synthesized CAT transcripts in the presence of streptomycin (A) or neomycin (B). Cell-free translation reactions were performed using S30 extracts prepared from N3433 and N433*rng* cells in addition to CAT mRNA. The protein samples were analyzed by 12% SDS-PAGE, and CAT protein production was detected using a phosphorimager. (**C**) The stability of CAT mRNA transcripts during *in vitro* translation. Cell-free translation reactions similar to those described above were performed using CAT mRNA transcripts uniformly labeled with [γ-^32^P]ATP. Samples were obtained after 1, 10, 20, 40 and 80 min of reaction time. The RNA transcripts were purified and analyzed on an 8% polyacrylamide gel containing 8 M urea. (**D** and **E**) Chemical probing of the 3' minor domain within the 16S rRNA with DMS in the presence of streptomycin. Chemical protection assays with DMS were performed using 30S subunits purified from the 70S ribosomes of the N3433 and N3433*rng* strains. Lanes 1–4, dideoxy sequencing reactions; lanes 5 and 9, control extension reaction with unmodified rRNA (no DMS); lanes 6 and 10, chemical probing in the absence of antibiotics; lanes 7, 8, 11 and 12, chemical probing with DMS in the presence of streptomycin at the molar ratio indicated. Reverse transcriptase stops at the position before a modified nucleotide; therefore, the bands indicate a modification at the next base in the sequence. (**F**) Molecular ratios of streptomycin bound to 30S subunits extracted from N3433 and N3433*rng* strains. 30S subunit sample (each 0.015 nmol dose) treated with a dose of streptomycin at 0.03, 3 and 300 nmol was analyzed by mass spectrometry as described in Materials and methods and Supplementary Figure S6.

Because *E. coli* cells that accumulate more 16S+3∼7 are less sensitive to aminoglycoside antibiotics than wild-type cells, we hypothesized that ribosomes containing 16S+3∼7 may be less susceptible to these antibiotics than those containing mature 16S rRNA. To test this hypothesis, we performed cell-free translation assays using *E. coli* S30 extracts prepared from N3433 and N3433*rng* cells. The aminoglycosides neomycin and streptomycin were tested because they exerted distinct antibiotic effects on the growth of *rng-*deleted strains. mRNA transcripts encoding chloramphenicol acetyl transferase (CAT) were synthesized *in vitro*, and poly(G) tails 20 nt in length were added to their 3′ ends to enhance the stability of these transcripts (28). The transcripts were gel purified and added to the S30 extract-based reactions. In the presence of 0.1 ng of streptomycin or 0.02 ng of neomycin per 1 μg of S30, the production of CAT from the N3433*rng*-derived S30 extract increased by 14% or 25%, respectively, compared with the production of CAT from the N3433-derived S30 extract (Figure 3A and B). It should be noted that the N3433*rng*-derived S30 extracts did not contain RNase G, whereas the N3433 extract still contained RNase G. As the lack of RNase G could have potentially increased the stability of the CAT mRNA transcripts used for *in vitro* translation and, consequently, the yield of protein synthesized, we tested the stability of the CAT mRNA transcripts in the reaction mixtures. The transcripts were labeled with [γ-^32^P]ATP and added to *in vitro* translation reactions under the same conditions described above. The mRNA samples were purified from the reaction mixtures at different time intervals and analyzed on an 8% polyacrylamide gel containing 8 M urea, appearing to decay at similar rates (Figure 3C). We obtained analogous results when RNA transcripts encoding the α fragment of β-galactosidase (LacZα) were used in these experiments (Supplementary Figure S4A–C). Next, we measured streptomycin-induced misincorporation of amino acids for N3433 and N3433*rng*-derived S30 extracts to test whether the results from cell-free translation assays are associated with direct effects of streptomycin on ribosomes. When S30 extracts were examined for the stimulation of the incorporation of isoleucine directed by poly(U) in the presence of streptomycin, those from N3433*rng* showed a decrease in the incorporation of isoleucine by 25% in the presence of streptomycin compared with those from N3433 (Supplementary Figure S4D), indicating that ribosomes from N3433*rng* restricted the response to streptomycin to a lesser extent. In addition, ribosomes purified from N3433*rng* were more resistant to streptomycin-induced conformational changes compared with those from N3433 when programmed with a fragment of the phage T4 gene 32 mRNA and having deacylated 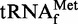 and tRNA^Phe^ in the P and A sites, respectively, in toeprinting assays (35) (Supplementary Figure S4E). Taken together, these results suggest that ribosomes in the *rng*-deletion strain are indeed more resistant to both neomycin and streptomycin than those in wild-type cells.

We further examined whether addition of purified RNase G into S30 extracts prepared from N3433*rng* can process 16S+3∼7 into mature 16S rRNA, and this alteration affects protein synthesis in the presence streptomycin. RNase G-treated S30 extracts of N3433*rng* had 16S rRNA precursors containing 3–6 extra nt at the 5′ end, rather than 16S+3∼7 (Supplementary Figure S4F), and showed the protein synthesis ability similar to that of N3433*rng*-derived S30 extracts treated with BSA in the presence of 0.1 ng of streptomycin per 1 μg of S30 (Supplementary Figure S4G). These results indicate that complete processing of the 5′-end of 16S rRNA by RNase G occurs during the assembly of ribosome.

### The presence of 3–7 extra nt at the 5′-end of 16S rRNAs reduces binding of aminoglycosides to 30S ribosome

It has been shown that certain nucleotide substitutions or the presence of an extra 66–115 nt at the 5′ end of the 16S rRNA can affect the interaction between 70S ribosomes and aminoglycoside antibiotics (8,26,36–40). Our results above also imply that the extra 3–7 nt at the 5′ end of the 16S rRNA in the *rng*-deletion cells may modulate the binding capacity for aminoglycosides. Accordingly, we compared the degree of protection of streptomycin-binding sites by wild-type 30S subunits with 30S subunits containing 16S+3∼7; the 30S subunits were prepared from 70S ribosomes isolated from N3433 and N3433*rng* strains. The subunits were incubated with streptomycin under the conditions used to reconstitute active 30S subunits from 16S rRNA and ribosomal proteins (41) and probed with DMS, which methylates adenine at N-1, cytosine at N-3 and guanine at N-7. When streptomycin was incubated with the 30S subunits containing 16S+3∼7, the 3′ minor domain was weakly protected by streptomycin compared with the wild-type 30S subunit. In the presence of a 40 000:1 molecular ratio of streptomycin to 30S subunits, methylation at residues A1492 and C1469 of 16S+3∼7 from the *rng*-deletion strain was increased by 33% and 32%, respectively (Figure 3D and E). We further tested neomycin in similar experiments and found that methylation at residue A1408 was increased by 7% in the presence of a 20:1 molecular ratio of neomycin to 30S subunits (Supplementary Figure S5). The identified nucleotide positions are known to be located in the decoding center targeted by both streptomycin and neomycin (29,42–44). To determine whether the results from chemical protection assays are related to lower binding capacity of N3433*rng*-derived 30S subunits to aminoglycosides, molecular ratios of streptomycin bound to 30S subunits were compared. Mass spectrometry analysis on 30S subunits purified from N3433 and N3433*rng* cells at various doses of streptomycin *in vitro* showed a lowered binding capacity of N3433*rng*-derived 30S subunits to streptomycin (Figure 3F and Supplementary Figure S6). The molecular ratios were varied depending on the streptomycin dose within 2- to 20 000-fold relative to the dose of 30S subunits. It was calculated that N3433*rng*-derived 30S subunits have the decreased streptomycin binding by 27–48%. These results indicate that the extra 3–7 nt at the 5′-end of 16S rRNA renders the decoding center less accessible to aminoglycosides.

### RNase G expression is downregulated by increased RNase III activity on *rng* mRNA in *E. coli* on exposure to aminoglycosides

**Figure 4.**
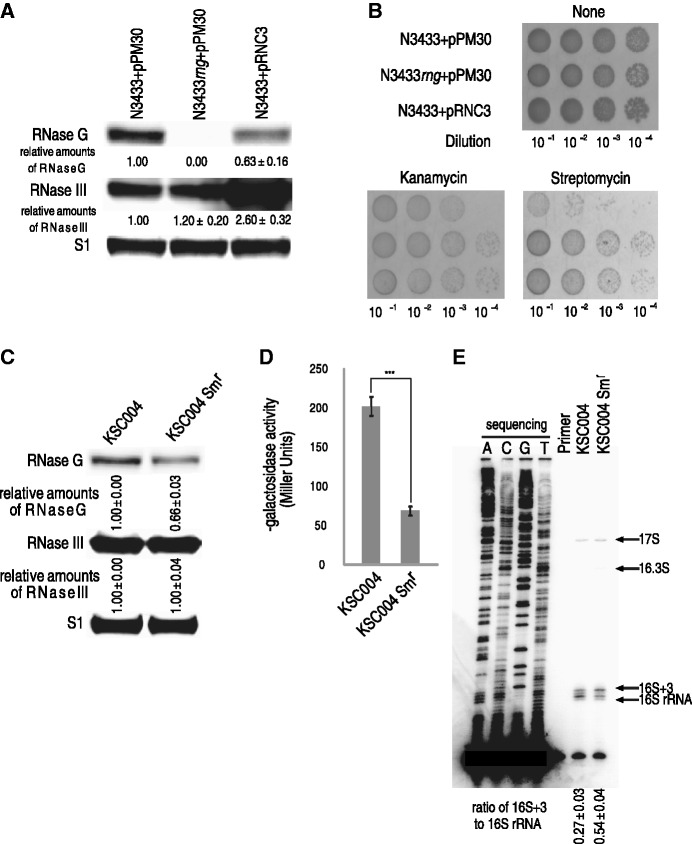
Downregulation of RNase G expression by increased RNase III activity. (**A**) Downregulation of RNase G expression by overexpression of RNase III. The cellular levels of RNase G and RNase III in N3433+pPM30, N3433*rng*+pPM30 and N3433+pRNC3 were estimated by a western blot analysis. (**B**) Effects of overexpression of RNase III on susceptibility to aminoglycosides. Strains N3433+pPM30, N3433*rng*+pPM30 and N3433+pRNC3 were grown till the early log phase in the presence of 1 mM IPTG to induce the transcription of *rnc* from a plasmid-borne promoter. When cells exponentially grew at 37°C, the serial 10-fold dilutions from 10^−1^ to 10^−4^ were spotted onto LB agar plates containing 1 mM IPTG and streptomycin (2 μg/ml) and incubated for 12 h at 37°C. (**C**) A western blot analysis of RNase G and RNase III in the streptomycin-resistant KSC004 isolate. Total protein was prepared from KSC004 cells that were not selected for streptomycin resistance and isolates of KSC004 that showed naturally induced streptomycin resistance (KSC004Sm^r^). Naturally induced streptomycin-resistant isolates of KSC004 were obtained as described in Materials and Methods. (**D**) Downregulation of RNase III activity by exposure of KSC004 to streptomycin. A β-galactosidase assay was performed as described in Materials and Methods. (**E**) Effects of increased RNase III activity on the 5'-end processing of 16S rRNA. Total RNA was isolated from KSC004 and KSC004Sm^r^ cells and analyzed as described in the legend of Figure 2A.

Given the experimental results indicating that the aminoglycoside-resistant isolates had reduced RNase G expression, which in turn causes the accumulation of partially unprocessed 16S rRNAs (16S+3), thereby conferring antibiotic resistance, we questioned the means by which *rng* expression is regulated in the aminoglycoside-resistant isolates. We previously noted that the steady-state levels of *rng* mRNA are downregulated by increased cellular RNase III concentrations based on genome-wide analyses of mRNA abundance in *E. coli* cells expressing different levels of RNase III (22). A semi-quantitative reverse transcription PCR (RT-PCR) analysis of *rng* mRNA in wild-type and *rnc*-deleted N3433 cells confirmed the microarray results (Supplementary Figure S7A), and an increased abundance of *rng* mRNA in *rnc*-deleted N3433 resulted in an approximately 4-fold increase in the amount of RNase G protein compared with that in the wild-type strain (Supplementary Figure S7B). Consistent with these results, the overexpression of exogenous RNase III in N3433 resulted in a 37% decrease in the amount of RNase G protein and, consequently, rendered the *E. coli* cells more resistant to kanamycin and streptomycin (Figure 4A and B). Based on this observation, we examined whether aminoglycoside-resistant isolates of N3433 had altered levels of RNase III expression. However, a western blot analysis revealed no significant alterations in the level of RNase III in these isolates (Supplementary Figure S8). It has been reported that RNase III activity, but not the protein level, can be modulated under bacteriophage infection, osmotic or cold-shock stress conditions (22,45,46). Therefore, we measured the enzymatic activity of RNase III in aminoglycoside-resistant *E. coli* (strain KSC004) isolates under aminoglycoside stress. This strain contains an RNase III target site in a single copy of a *pnp′–′lacZ* reporter gene fusion (20,47), the expression of which is controlled by RNase III cleavage in the *pnp* untranslated leader; ß-galactosidase production from this fusion construct is increased ∼3-fold in cells depleted for RNase III activity (48). First, we isolated KSC004 *E. coli* cells with naturally induced resistance to streptomycin using the methods described above to obtain aminoglycoside-resistant N3433 isolates. Consistent with the experimental results obtained from the aminoglycoside-resistant isolates of N3433, the RNase G expression levels were decreased by ∼34% compared with their levels in wild-type KSC004 (Figure 4C). Although the RNase III expression levels in the streptomycin-resistant isolates of KSC004 were similar to wild-type, the ß-galactosidase activity was decreased by ∼3-fold (Figure 4D). Again, when cultured in the presence of streptomycin, these isolates accumulated approximately two times more 16S+3 compared with the same isolates cultured in the absence of streptomycin (Figure 4E), indicating a correlation between aminoglycoside resistance and 16S+3 accumulation.

The experimental results above indicate that RNase III may control the degradation of *rng* mRNA. Indeed, *in vitro* cleavage assays with affinity-purified RNase III protein and *rng* mRNA transcripts and primer extension analyses using *rng* mRNA clearly demonstrated that RNase III cleaves the coding region of *rng* mRNA (Supplementary Figure S9). Moreover, the introduction of base substitutions at the identified RNase III sites resulted in enhanced RNase G expression (Supplementary Figure S9). Based on these results, we conclude that RNase III directly cleaves the coding region of *rng* mRNA and, consequently, controls the expression of RNase G.

### Effects of *rng*-deletion on 16S rRNA processing and aminoglycoside resistance in *Salmonella* enterica Typhimurium SL1344

Homologs of both RNase III and RNase G are present in *E. coli*, *S. enterica* serovar Typhimurium*, Klebsiella pneumoniae*, *Shigella dysenteriae*, *Shigella flexneri* and *Shigella sonnei*. Among these, *S. enterica* is physiopathologically different from the others. Therefore, *S. enterica* serovar Typhimurium strain SL1344 was chosen to validate the causal relationship between aminoglycoside resistance and incomplete 16S rRNA processing by RNase G in related gram-negative bacteria. First, we constructed an *rng*-deleted *S. enterica* SL1344 strain (SL1344*rng*). The absence of the RNase G protein in SL1344*rng* was confirmed by a western blot analysis using polyclonal antibodies against *E. coli* RNase G (Supplementary Figure S10A), and a high degree of homology (97% amino acid identity) between these RNase G proteins allowed the efficient detection of RNase G in *S. enterica* SL1344. There was no detectable growth defect in the SL1344*rng* cells when grown in a rich medium (Supplementary Figure S10B). Next, we tested the susceptibility of SL1344*rng* cells to kanamycin and paromomycin by spotting serially diluted cultures on an LB agar plate containing 5 μg/ml kanamycin or 7 μg/ml paromomycin; wild-type cells were also included. The results showed a marked increase in the resistance of the SL1344*rng* cells to kanamycin and paromomycin compared with the wild-type cells (Supplementary Figure S10B). The primer extension analysis of the 5′ end of the 16S rRNA from the SL1344*rng* cells showed the accumulation of precursor 16S rRNA species with sizes similar to those of the *E. coli* 16S rRNA precursors (Supplementary Figure S10C). These results reveal that the susceptibility of *S. enterica* cells to aminoglycosides is also affected by the extent of RNase G-mediated 16S rRNA processing. This finding implicates that RNase G can function as a key regulator of aminoglycoside resistance in *E. coli* and related gram-negative bacteria.

## DISCUSSION

The *rng* gene is regarded as a nonessential gene in *E. coli*, as indicated by the finding that *rng*-deleted cells do not exhibit a growth defect (17). This observation led to the hypothesis that ribosomes can function normally, regardless of 16S rRNA processing by RNase G. Consistent with this hypothesis, we also found that ribosomes in *rng*-deleted cells containing incompletely processed 16S rRNA (16S+3∼7) synthesize target proteins in a cell-free translation system as efficiently as ribosomes from wild-type cells (Figure 3A–C; Supplementary Figure S4).

Although ribosomes in *rng*-deleted cells perform protein synthesis as efficiently as those from wild-type cells, we found that the *rng*-deletion strain that accumulated 16.3S and 16S+3∼7 in 70S ribosomes was more adaptable to aminoglycosides compared with wild-type (Figure 1B and C and 2A and B). The adaptive resistance phenotype in *E. coli* cells that accumulate 16S rRNA precursors appears to result from the lower binding capacity of ribosomes containing 16S rRNA precursors for aminoglycosides. This finding is based on the results of DMS chemical probing and mass spectrometry analysis, which revealed that ribosomes containing unprocessed 16S rRNA had a lower binding capacity for aminoglycosides (Figure 3D–F; Supplementary Figures S5 and S6). Furthermore, these results are consistent with the results from cell-free translation assays (Figure 3A–C; Supplementary Figure S4). Our findings reveal that the *rng*-deletion strain ribosomes and those from *E. coli* isolates that acquired aminoglycoside resistance either transiently or by mutagenesis at the RNase G cleavage site of 16S rRNA contain 16S rRNA precursors with an extra 3–8 nt at the 5′ terminus, indicating that these ribosomes are responsible for the aminoglycoside resistance phenotype of these *E. coli* strains. These results imply that a 16S rRNA containing an additional 3–8 nt at the 5′-terminus induces conformational changes in ribosomes, thereby influencing the binding of aminoglycosides. In support of our hypothesis, a recent report showed that the unprocessed 5′ end of 16S rRNA causes an alternative structure in helix 1 (8).

Our results contrast with a previous study by Roy-Chaudhuri *et al.* (8). The study showed that an *E. coli* strain deleted for the *rng* gene (MG1655*rng*) is more susceptible to neomycin and paromomycin compared with the parental strain (MG1655), a phenotype thought to be due to the presence of 16.3S rRNA in ribosomes. For this reason, we obtained MG1655 and MG1655*rng* strains from Dr. Gloria M. Culver′s group that have been used for experimental results shown in Figure 3C and D in the corresponding paper. We found that the strains obtained from Dr. Gloria M. Culver′s group appear to contain antibiotic-resistant genes, as their MG1655 and MG1655*rng* exhibited resistance to high concentrations of kanamycin (100 μg/ml) and chloramphenicol (20 μg/ml), respectively. (Supplementary Figure S11). It is unknown whether the disparate conclusions that Roy-Chaudhuri *et al.* and our group obtained result from differences in the methods and/or source to obtain these *E. coli* strains. However, it should be addressed that the original genotype of MG1655 does not confer resistance to kanamycin at 100 μg/ml, and it is better to use an MG1655*rng* strain, however it was constructed, that does not have an antibiotic resistance marker that confers resistance to especially those targeting ribosome. While we conclude that the strains (MG1655 and MG1655*rng*) used in Roy-Chaudhuri *et al.* should be reevaluated, our experimental results from two independent *E. coli* stains that are deleted for *rng* (MG1655*rng* and N3433*rng*), as well as *E. coli* isolates that acquired aminoglycoside resistance either transiently or by mutagenesis at the RNase G cleavage site of 16S rRNA, consistently showed more resistance to aminoglycosides compared with wild-type *E. coli* strains. Our biochemical analyses on ribosomes from *rng*-deleted *E. coli* strains that include cell-free translation assays, DMS chemical probing and mass spectrometry analysis clearly supported our genetic data. In addition, another bacterial species related to *E. coli*, *S. enterica* SL1344 strain, developed the aminoglycoside-resistance phenotype when the *rng* gene was deleted. We think that the simplest interpretation of our findings is that accumulation of 16S rRNA precursors containing an extra 3–8 nt at the 5’-terminus in ribosomes in *E. coli* cells is responsible for the aminoglycoside-resistance phenotype.

The proteome profiles of wild-type *E. coli* cells that had transiently acquired ‘noninherited resistance’ to aminoglycoside antibiotics showed that aminoglycoside resistance is most strongly associated with decreased expression levels of RNase G and altered, albeit less significantly, expression levels of ribosomal subunits and associated proteins. This result suggests a possibility that altered expression levels of ribosomal subunits and associated proteins may contribute to the phenotype of aminoglycoside resistance. This notion further suggests that a decrease in the capacity of ribosomes containing partly unprocessed 16S rRNA to bind aminoglycosides might be caused by altered composition and/or stoichiometry of the ribosome particles. While this possibility is still open, we were not able to detect such changes in 30 subunits isolated from *rng*-deleted *E. coli* cells compared with those from wild-type cells (Supplementary Figure S6D).

Our primer extension data showed that mature 16S rRNA was formed in the ribosomes of *rnc*-deletion or RNase E-depleted cells (KSL2000) but did not form in the *rng*-deletion strain (Supplementary Figure S12) as seen in previous studies (10,14,15). This result suggests that the 5′ maturation of 16S rRNA by RNase III and RNase E can be accomplished by other ribonucleases through unknown pathways. Conversely, the maturation of the 16S rRNA 5′ end by RNase G cannot be accomplished by other processing pathways, leaving an extra 3–7 nt. Therefore, RNase G is a unique ribonuclease that is absolutely required for the complete 5′-end maturation of 16S rRNA.

The accumulated data, both previous and present, provide indirect evidence for the importance of RNase G (14–16,19). Considering that RNase G proteins are as abundant as RNase E (2–3 × 10^4^ proteins/cell) (Supplementary Figure S13), which plays a key role in the degradation of a majority of mRNAs and processing of such structural RNAs as rRNAs, tRNAs and M1 RNAs (49–54), RNase G may have significant physiological roles that have not yet been identified. One such example is its potential role in forming cytoskeleton-like structures known as cytoplasmic axial filaments (55), the function of which remains unknown. Our present study also emphasizes the physiological importance of RNase G in inducing aminoglycoside resistance in *E. coli* and related gram-negative bacteria.

**Figure 5.**
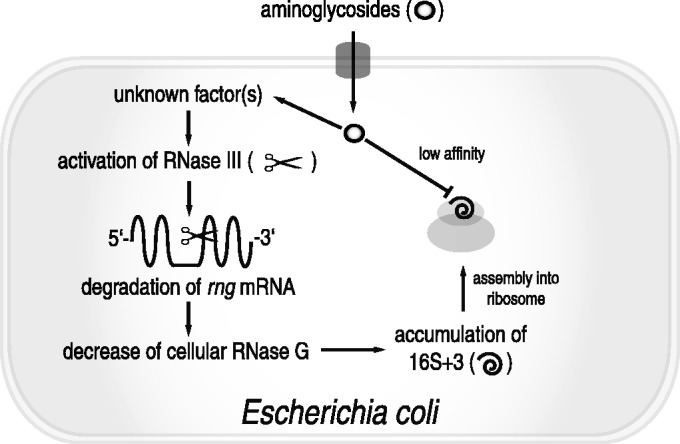
A model for the adaptive resistance of *E. coli* to aminoglycoside antibiotics via antibiotic stress-induced sequential modulation of the endoribonucleolytic activity of RNase III and RNase G. An unknown signal triggered by the exposure of *E. coli* to aminoglycosides activates RNase III, promoting the digestion of *rng* mRNA, which leads to decreased cellular expression of RNase G. Owing to the reduction in RNase G expression, processing of the 5' terminus of 16S rRNA is impaired, resulting in the accumulation of 16S+3. 16S+3 is normally assembled into the 30S subunit and eventually complexes with the 50S subunit, forming a functional 70S ribosome. This 70S ribosome has decreased binding capacity for aminoglycoside antibiotics, consequently resulting in aminoglycoside resistance in *E. coli*.

Although the exact physiological cue required to generate incompletely processed 16S rRNA by the downregulation of RNase G associated with increased RNase III activity remains unknown (Figure 5), we conclude that the modulation of *rng* expression plays an important physiological role in the stress response to aminoglycoside antibiotics. Additionally, this modulation may contribute to the development of noninherited resistance to aminoglycosides in certain bacterial species. Based on our findings, we suggest that a mechanism involving the downregulation of RNase G expression may contribute to the occurrence of certain bacterial species that are resistant to low levels of aminoglycosides in living systems.

## SUPPLEMENTARY DATA

Supplementary Data are available at NAR Online.

## FUNDING

National Research Foundation of Korea [2010-0029167 to Y.L., 2011-0028553 to K.L.]; Next-Generation BioGreen 21 Program, Rural Development Administration, Republic of Korea [SSAC, PJ009025 to K.L.]; Marine and Extreme Genome Research Center Program of the Ministry of Oceans and Fisheries, Republic of Korea [to J.H.L]; Chung-Ang University Excellent Student Scholarship [(partially) to S.H.]. Funding for open access charge: National Research Foundation of Korea [2011-0028553].

*Conflict of interest statement*. None declared.

## Supplementary Material

Supplementary Data
