# Supplementary material for: Antibiotic stress-induced modulation of the endoribonucleolytic activity of RNase III and RNase G confers resistance to aminoglycoside antibiotics in Escherichia coli
Source: Nucleic Acids Res. 2014 Jan 30;42(7):4669–81. doi: 10.1093/nar/gku093 (PMC3985665; doi:10.1093/nar/gku093)
Supplement: Supplementary Data [file supp_42_7_4669__index.html]

Antibiotic stress-induced modulation of the endoribonucleolytic activity of RNase III and RNase G confers resistance to aminoglycoside antibiotics in Escherichia coli — Antibiotic stress-induced modulation of the endoribonucleolytic activity of RNase III and RNase G confers resistance to aminoglycoside antibiotics in Escherichia coli — Supplementary Data 

# Antibiotic stress-induced modulation of the endoribonucleolytic activity of RNase III and RNase G confers resistance to aminoglycoside antibiotics in *Escherichia coli*

## Supplementary Data

files

**Files in this Data Supplement:**

- Supplementary Data - pdf file
