# Supplementary material for: Antibiotic stress-induced modulation of the endoribonucleolytic activity of RNase III and RNase G confers resistance to aminoglycoside antibiotics in Escherichia coli
Source: Nucleic Acids Res. 2014 Jan 30;42(7):4669–81. doi: 10.1093/nar/gku093 (PMC3985665; doi:10.1093/nar/gku093)
Supplement: Supplementary Data [file supp_gku093_nar-02507-z-2013-File007.pdf]

Supplementary data

Figure S1. Effects of *rng* deletion on the susceptibility of *E. coli* to aminoglycoside antibiotics. Growth characteristics of N3433 and N3433*rng* strains in LB media containing kanamycin, neomycin, paromomycin, or streptomycin. Overnight cultures grown in LB were diluted 1:100 with LB medium containing each of the antibiotics. The growth of these cultures was monitored by measuring their cell density (OD_{600}) at the indicated time points. The concentrations of antibiotics were same as those seen in Figure 1E in the main text.

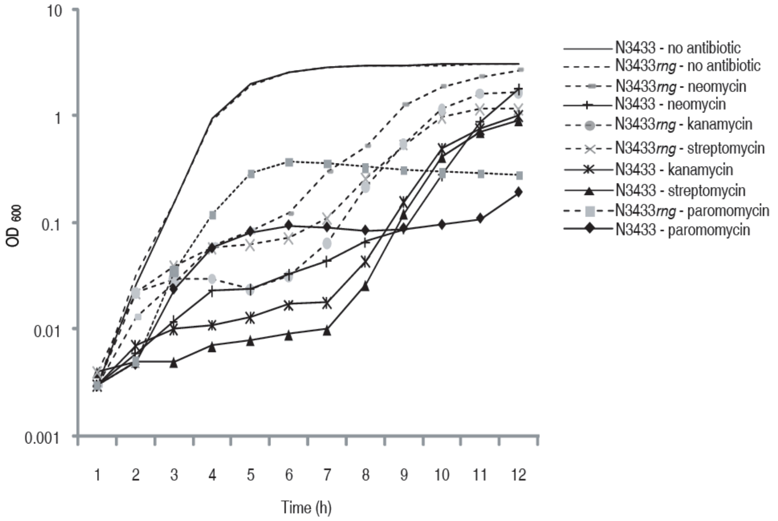

Figure S2. Effects of *rng* deletion on 16S rRNA 5' end processing in *E. coli* strain MG1655. Total RNA, 30S subunits, and 70S ribosomes were purified from MG1655 and MG1655*rng* strains and used for primer extension analysis.

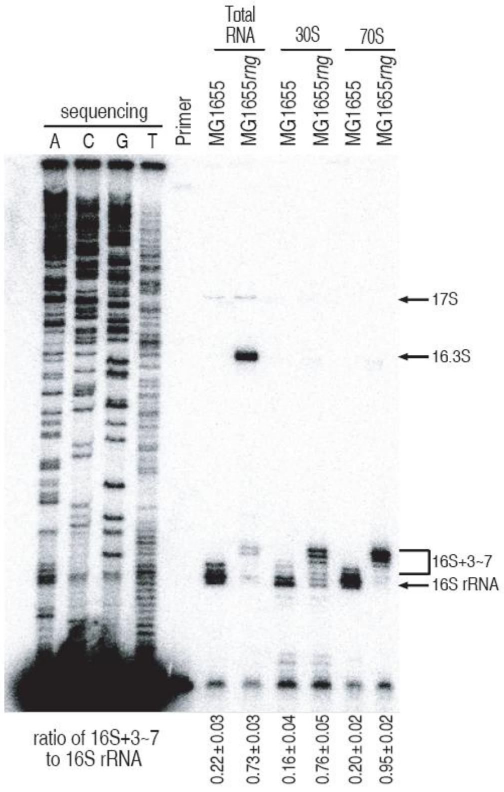

Figure S3. Effects of insertion of three nucleotides in the RNase G cleavage site on aminoglycoside resistance and 16S rRNA 5' end processing. (A) Sequence analysis of seven clones that grew better than N3433-pRNA9 on LB media containing 2 μg/ml streptomycin and 1 mM IPTG. **(B)** Primer extension analysis of the 5' end of the 16S rRNA from RNase G cleavage site mutants. Mutants with insertion of 3 nucleotides in Fig. S4A were constructed with the corresponding p-RNA9 clones. **(C)** Effects of a 3nt insertion in the 16S rRNA RNase G cleavage site on the susceptibility of *E. coli* to aminoglycosides. Strains N3433+pRNA9, N3433*rng*+pRNA9, and N3433+pRNA9-three random nt insertion mutations were grown to early log phase (OD_{600}=0.1), and 1 mM IPTG was added to induce the transcription of *rrn*B from a plasmid-borne promoter. The cells were further grown to mid-log phase (OD_{600}=0.6~0.7) at 37°C. Serial 10-fold dilutions of cells from 10^{-1} to 10^{-4} were spotted onto LB agar plates containing kanamycin (2 μg/ml), neomycin (1 μg/ml), paromomycin (5 μg/ml), or streptomycin (2 μg/ml), and incubated in the presence of 1 mM IPTG for 12 h at 37°C.

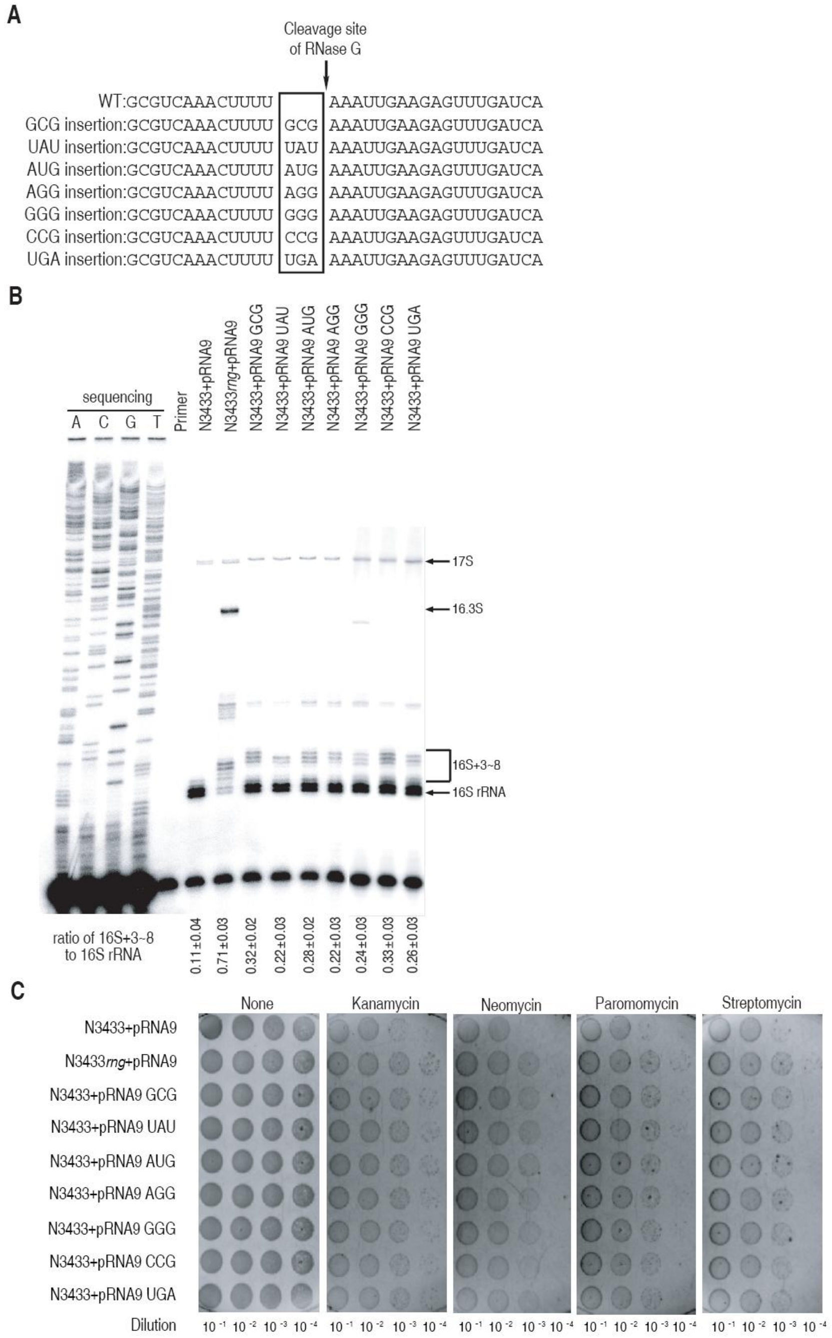

Figure S4. Effects of 16S+3~7 accumulation on the ability of 70S ribosomes to synthesize LacZα protein in the presence of streptomycin or neomycin. (A) *In vitro* translation of the LacZα protein using *in vitro*-synthesized *lacZ*α transcripts in the presence of streptomycin **(A)** or neomycin **(B)**. Cell-free translation reactions were performed using S30 extracts prepared from N3433 and N433*rng* cells in addition to *lacZ*α mRNA. Proteins were separated by 13% Tricine-SDS-PAGE, and LacZα protein was detected using a phosphorimage analyzer. **(C)** Stability of *lacZ*α transcripts during *in vitro* translation. Cell-free translation reactions, similarly as described above, were performed using *lacZ*α mRNA transcripts uniformly labeled with [γ-^{32}P]ATP. Samples were obtained after 1, 10, 20, 40, and 80 min reaction time. The labeled transcripts were purified and analyzed on a 10% polyacrylamide gel containing 8 M urea. **(D)** Streptomycin-induced misincoporation of amino acids. Ratios of [^{3}H]isoleucin incorporation under the direction of poly(U) in the presence of streptomycin were measured using the procedure as described previously (1,2), except that S30 extracts was used in place of purified ribosome subunits. Ratios of [^{3}H]isoleucin incorporation by N3433*rng*-derived S30 extracts and N3433-derived S30 extracts in the presence of streptomycin were normalized by setting those by N3433-derived S30 extracts in the absence of streptomycin as one **(E)** Toeprinting analysis of defined ribosome complexes in the presence of streptomycin. The toeprinting assay was performed as described previously (3), except that binding buffer (70 mM HEPES [pH 7.8], 20 mM $MgCl_2$, 300 mM KCl, 0.5 mM EDTA, and 2 mM DTT) was used in place of the buffer (80 mM potassium cacodylate (pH 7.2), 20 mM magnesium acetate, 150 mM ammonium chloride). Ribosomes were programmed with a fragment of T4 gene 32 mRNA and contained $tRNA_f^{Met}$ and $tRNA^{Phe}$ in the P and A sites, respectively. In the presence of streptomycin, a toeprint signal at position +17 accumulates. Tight-couple 70S ribosomes were isolated from N3433 and N3433*rng* strains as described in the Materials and Methods. **(F)** Effects of RNase G addition into S30 extracts on the processing of 16S rRNA+3~7. Approximately 25 µg of S30 extracts were incubated with 1 µg of purified RNase G for 30 min at 37 °C. Bovine serum albumin (1 µg BSA) was included as an experimental control under the same reaction conditions. The cleavage products were purified by phenol extraction, precipitated with ethanol, and analyzed by primer extension, as described in the Materials and Methods. **(G)** Effects of RNase G addition into S30 extracts on the protein synthesis. Cell-free translation reactions were performed, as described above, using CAT mRNA transcripts and S30 extracts treated with purified RNase G. The S30 extracts treated with BSA were used as an experimental control. Proteins were separated by 12% SDS-PAGE, and CAT protein production was detected using a phosphorimage analyzer. The significant differences as revealed by one way ANOVA ($P<0.05$) are indicated by different letters.

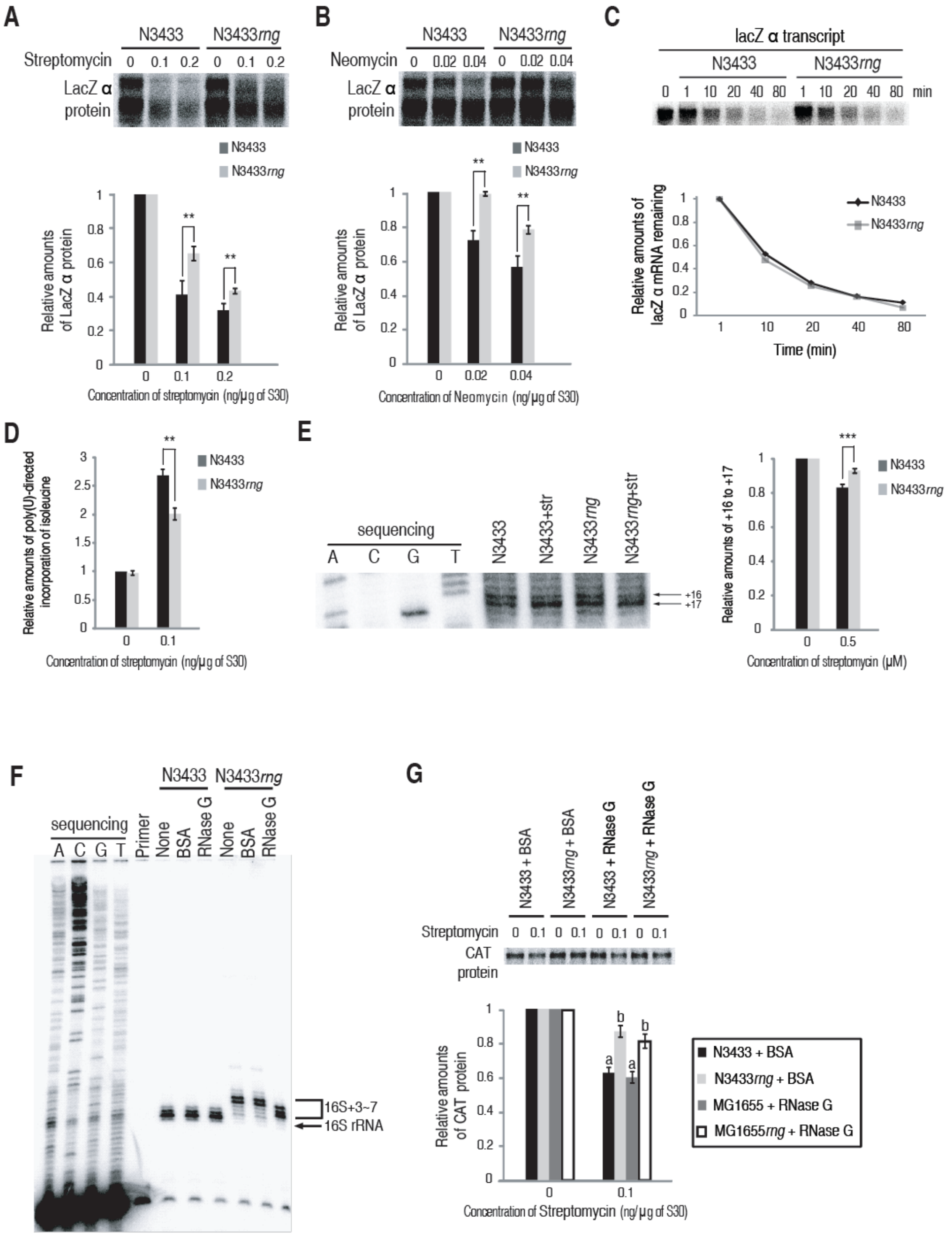
A
N3433
N3433rng
Streptomycin 0 0.1 0.2 0 0.1 0.2
LacZ α protein
N3433
N3433rng
Relative amounts of LacZ α protein
Concentration of streptomycin (ng/μg of S30)
B
N3433
N3433rng
Neomycin 0 0.02 0.04 0 0.02 0.04
LacZ α protein
Relative amounts of LacZ α protein
Concentration of Neomycin (ng/μg of S30)
C
lacZ α transcript
N3433
N3433rng
0 1 10 20 40 80 1 10 20 40 80 min
Relative amounts of lacZ α mRNA remaining
Time (min)
D
Relative amounts of poly(U)-directed incorporation of isoleucine
Concentration of streptomycin (ng/μg of S30)
E
sequencing
A C G T
N3433
N3433+str
N3433rng
N3433rng+str
+16
+17
Relative amounts of +16 to +17
Concentration of streptomycin (μM)
F
N3433
N3433rng
sequencing
A C G T
Primer
None
BSA
RNase G
16S+3~7
16S rRNA
G
N3433 + BSA
N3433rng + BSA
N3433 + RNase G
N3433rng + RNase G
Streptomycin 0 0.1 0 0.1 0 0.1 0 0.1
CAT protein
Relative amounts of CAT protein
N3433 + BSA
N3433rng + BSA
MG1655 + RNase G
MG1655rng + RNase G
Concentration of Streptomycin (ng/μg of S30)

Figure S5. Chemical probing of the 3' minor domain within 16S rRNA with dimethyl sulfate (DMS) in the presence of neomycin. Chemical protection assays with DMS were performed as described in Fig. 3D and E, except that neomycin was used in place of streptomycin. Lanes 1 to 4, dideoxy sequencing reactions; lanes 5 and 9, control extension reaction with unmodified rRNA (no DMS); lanes 6 and 10, chemical probing in the absence of neomycin; lanes 7, 8, 11, and 12, chemical probing with DMS in the presence of neomycin at the molar ratio indicated. Reverse transcriptase stops at the position before a modified nucleotide; therefore, the bands indicate a modification at the next base in the sequence.

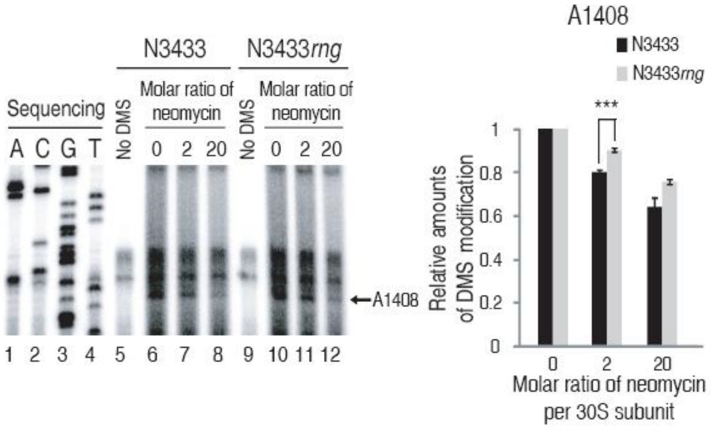

Figure S6. Mass spectrometric analysis of aminoglycosides. (A) Standard curves for quantification of streptomycin dose in a range of 0.015 to 15 nmol with a mixture of 1.5 nmol either of amikacin, gentamicin, or kanamycin used as internal standard for calculation of relative signal intensity of streptomycin (STR). **(B)** A total ion chromatogram between *m/z* 470 and 590 showing relative abundance of monoisotopic mass ion peak of each 1.5 nmol dose of amikacin (AMK, *m/z* = 586.29), gentamicin (GEN, *m/z* = 478.32), kanamycin (KAN, *m/z* = 485.25), and streptomycin (STR, *m/z* = 582.27). **(C)** Merged plots of total ion chromatograms between *m/z* 580 and 590 showing relative levels of monoisotopic ion peaks of STR in a range of 0.015 to 15 pmol with the corresponding internal standard peak of AMK expressed as 100. **(D)** Protein band intensities of 30S subunit proteins of N3433 (lanes 2 to 6) and N3433*rng* (lanes 7 to 11) stained with Coomassie Brilliant R250 before and after application onto protein desalting G25 columns: lane1, blank; lanes 2 and 7, 30S subunit bands before the desalting; and lanes 4 to 6 and 8 to 11, 30S subunit bands after the desalting. Ratio of streptomycin dose to 30S subunit dose: lanes 3 to 8, 0:1; lanes 4 and 9, 2:1; lanes 5 and 10, 200:1; and lanes 6 and 11, 20000:1. Relative band intensities (SD, standard deviation) were estimated in comparison with lane 2 expressed as a unit by analysis of histogram peaks using Molecular Dynamics ImageQuant program version 5.2 **(E-H)** Tandem mass spectra assigned to molecular ion $[MH]^+$ and fragment ions generated from electrospray ionization collision-induced dissociation of amikacin **(E)**, gentamicin **(F)**, kanamycin **(G)**, and streptomycin **(H)**.

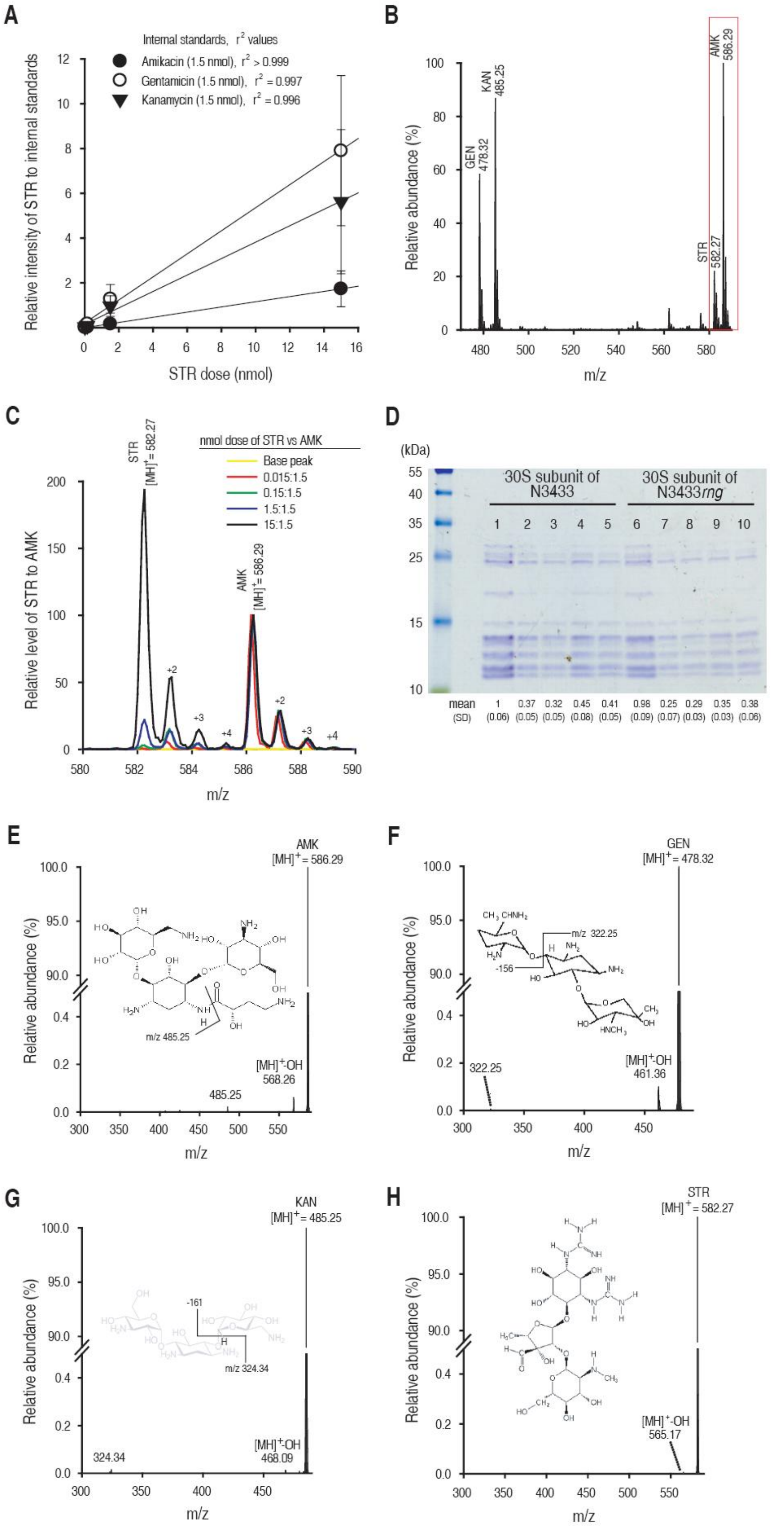
A
Internal standards, r2 values
Amikacin (1.5 nmol), r2 > 0.999
Gentamicin (1.5 nmol), r2 = 0.997
Kanamycin (1.5 nmol), r2 = 0.996
Relative intensity of STR to internal standards
STR dose (nmol)
B
Relative abundance (%)
GEN 478.32
KAN 485.25
STR 582.27
AMK 586.29
m/z
C
nmol dose of STR vs AMK
Base peak
0.015:1.5
0.15:1.5
1.5:1.5
15:1.5
STR [MH]+= 582.27
AMK [MH]+= 586.29
Relative level of STR to AMK
m/z
D
(kDa)
30S subunit of N3433
30S subunit of N3433rng
mean
(SD)
1 0.37 0.32 0.45 0.41 0.98 0.25 0.29 0.35 0.38
(0.06) (0.05) (0.05) (0.08) (0.05) (0.09) (0.07) (0.03) (0.03) (0.06)
E
AMK [MH]+= 586.29
m/z 485.25
[MH]+-OH 568.26
485.25
Relative abundance (%)
m/z
F
GEN [MH]+= 478.32
m/z 322.25
-156
322.25
[MH]+-OH 461.36
Relative abundance (%)
m/z
G
KAN [MH]+= 485.25
-161
m/z 324.34
324.34
[MH]+-OH 468.09
Relative abundance (%)
m/z
H
STR [MH]+= 582.27
[MH]+-OH 565.17
Relative abundance (%)
m/z

Figure S7. Negative regulation of *rng* mRNA by RNase III. (A) RT-PCR analysis to determine the cellular abundance of *rng* mRNA. Total RNAs were isolated from N3433, N3433*rnc*, and N3433*rng* and used for semi-quantitative RT-PCR experiments. The primers used for *rng* were 5'-GTGAGAAAAGGGATAAACAT-3' and 5'-TTACATCATTACGACGTCAA-3'. **(B)** Relative amounts of RNase G protein in the *rnc*-deletion strain. The cellular levels of RNase G and RNase III in N3433, N3433*rng*, and N3433*rnc* were estimated by a western blot analysis.

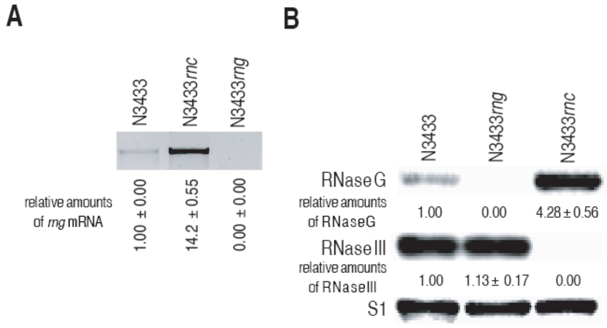

Figure S8. Relationship between RNase III expression levels and aminoglycoside resistance in *E. coli*. Cellular levels of RNase III in streptomycin-resistant (Sm^r) or kanamycin-resistant (Km^r) isolates and their revertants (Sm^s or Km^s) were estimated by western blot analysis. Expression levels of RNase III were nearly constant in aminoglycoside-resistant (Sm^r and Km^r) isolates and their revertants, whilst cellular levels of RNase G decreased in aminoglycoside-resistant (Sm^r and Km^r) isolates and returned to the wild-type levels, when cells were cultured in a medium without aminoglycosides, as described in the main text.

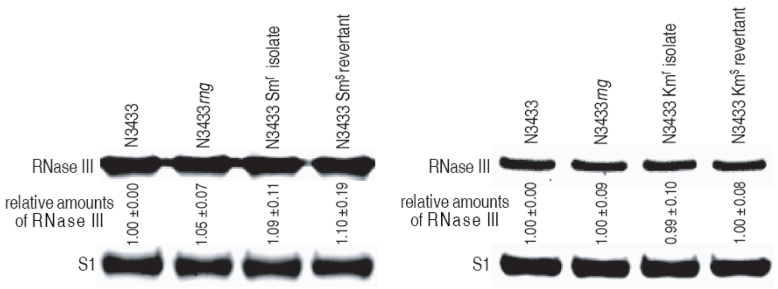

Figure S9. Identification of *rng* mRNA RNase III cleavage sites *in vitro* and *in vivo*.

(A) *In vitro* cleavage of *rng* mRNA. *In vitro* transcription was performed using the MEGAscript^{TM} T7 kit (Ambion, Inc.) according to the manufacturer's instructions with a PCR fragment containing the coding region of *rng* mRNA as a template and T7 RNA polymerase. The *rng* mRNA template was amplified from N3433 genomic DNA using a 5'-T7-rng primer (5'-TAATACGACTCACTATAGGGATGACGGCTGAATTGTTAGT-3') and a 3'-rng primer (5'-TCACATCATTACGACGTCAA-3'). The transcripts were labeled internally or at the 5'-end with [α-^{32}P]UTP or [γ-^{32}P]ATP, respectively. A 1-pmol sample of the 5' end- or internally-labeled *rng* mRNA was incubated with 0.5-1 μg of purified RNase III in the presence of 20 U of RNase OUT^{TM} Recombinant Ribonuclease Inhibitor (Invitrogen^{TM}) and 0.25 μg ml^{-1} of yeast tRNA in cleavage buffer at 37°C (4). RNase III proteins were purified from BL21(DE3)*rnc105 recA*::Tn9 cells harboring pET15b-rnc, as previously described (5). Samples were withdrawn at the indicated time intervals and separated on an 8% polyacrylamide gel containing 8 M urea. The cleavage products of different sizes are indicated with I, II, and III. The size markers were generated by 5' end-labeling using a Century marker (Ambion) with ^{32}P. Cleavage of the ^{32}P 5'-end-labeled *rng* mRNA with purified RNase III generated cleavage products with sizes of approximately 170 to 180 (I) and 120 to 130 (II) nt, whereas cleavage of the uniformly radiolabeled *rng* transcripts with RNase III produced one additional band with a size of approximately 40 to 60 (III) nt (marked with arrows). **(B, C)** Primer extension was performed with the primers rng+219R (5'-TCTTCATGCATCCGACATCATGCCG-3') **(B)** and rng+166R (5'-CCGCCTGCATACCCGGAAGT-3') **(C)**. Total RNAs were prepared from N3433 and N3433*rnc* cells that were grown to mid-log phase (OD_{600}=0.6). The cleavage sites are indicated with arrows. A primer extension analysis was performed using total RNA samples prepared from wild-type and *rnc*-deleted *E. coli* strains. Primer extension using the rng+219R primer showed that *rng* mRNA is efficiently cleaved between positions 172 and 173 **(B)**. The primer extension analysis using the rng+166R primer, which anneals to positions 147-166 of the *rng* coding sequence, identified a cleavage site in the 5' terminus located between positions 127 and 128 **(C)**. **(D)** The predicted secondary structure of *rng* mRNA. The secondary structure was deduced using the M-fold (http://mfold.rna.albany.edu) program. The RNase III cleavage sites of *rng* mRNA are indicated with arrows, as based on the results from (A), (B), and (C). The hairpin RNA containing the RNase III cleavage sites is shown in the right panel. **(E)** Effects of nucleotide substitutions at the identified cleavage sites of RNase III on the expression levels of RNase G. pRNG3 plasmids containing random nucleotide substitutions at the RNase III cleavage sites were constructed using a PCR fragment that was amplified with the primers rng-5' (5'-GGATCCGCGGCCGCTTTAAGAAGGAGATATACATATGAGAAAAGGGATAAAC-3') and rng213R-13N (5'-GGATGCATGAAGAAACGCGGCTTTATCCAGCCCAA$[N]_{13}$CGCCTGCATACCCGGAAGTAC-3'), digested with NotI and NsiI, and ligated into pRNG3. N3433*rng* cells were transformed with a population of pRNG3 plasmids containing random mutations, and clones overexpressing full-length RNase G were screened. A western blot analysis was performed on the clones expressing wild-type (N3433*rng*+pRNG3-WT) or mutant *rng* mRNAs (N3433*rng*+pRNG3-MT) containing nucleotide substitutions at positions 165-171 and 173-177 (^{165}AUCCGGCUUAUAC^{177}).

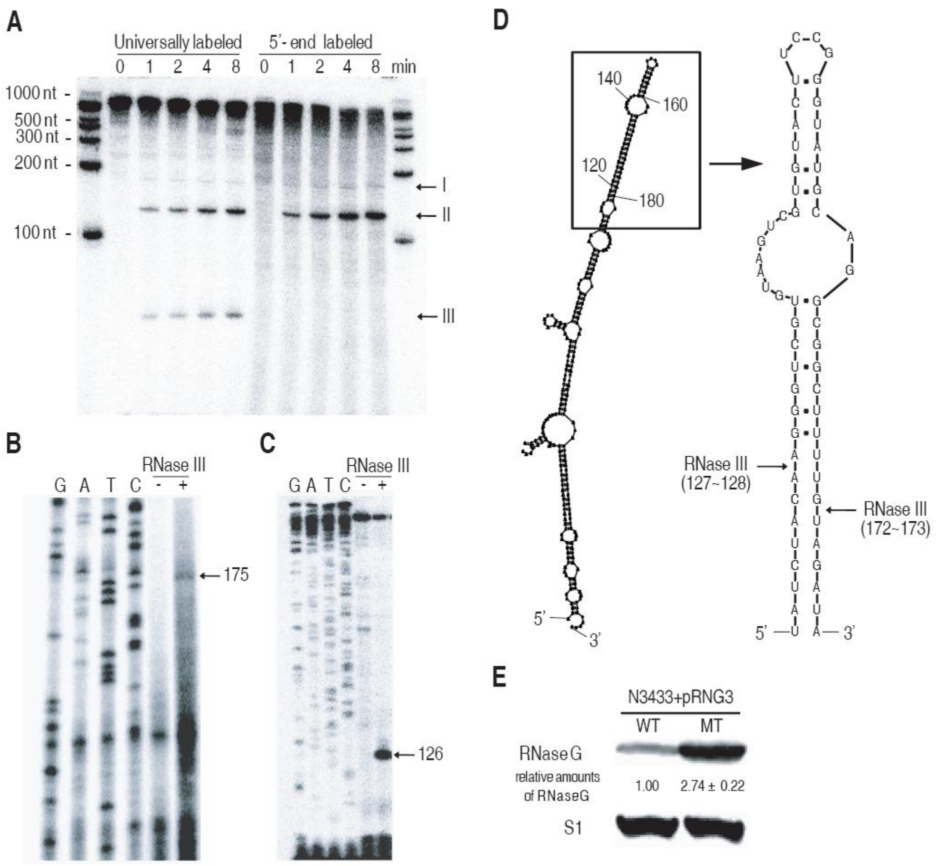

A
Universally labeled
5'- end labeled
0 1 2 4 8 0 1 2 4 8 min
1000 nt
500 nt
300 nt
200 nt
100 nt
I
II
III
B
G A T C
RNase III
- +
175
C
G A T C
RNase III
- +
126
D
140
160
120
180
5'
3'
RNase III
(127~128)
RNase III
(172~173)
E
N3433+pRNG3
WT
MT
RNase G
relative amounts
of RNaseG
1.00
2.74 ± 0.22
S1

Figure S10. Effects of *rng* deletion on 16S rRNA processing and aminoglycoside resistance in *Salmonella enterica* Typhimurium SL1344. (A) Western blot analysis of the *S. enterica* strain SL1344 deleted for *rng*. **(B)** Effect of *rng* deletion in *S. enterica* SL1344 on the susceptibility to kanamycin and paromomycin. Cells were grown to mid-log phase at 37°C, and diluted in a range of 10^{-1}–10^{-4} to spot 10 μl-aliquots onto LB agar plates containing kanamycin (5 μg/ml) or paromomycin (7 μg/ml), and then incubated for 12 h at 37°C. **(C)** Analysis of the 16S rRNA5' end in the *S. enterica* strain SL1344 deleted for *rng*. Total RNAs were isolated from cultures of *S. enterica* SL1344 harboring pACYC177 and *S. enterica* SL1344*rng* harboring pACYC177 or pRNG-ST, and were analyzed by primer extension analysis. *E. coli* strains N3433 and N3433*rng* were included as reference strains. The plasmid pRNG-ST contains a cloned copy of the *S. enterica rng* gene. To construct pRNG-ST, the *rng* gene of *S. enterica* was PCR amplified from *S. enterica* genomic DNA using the RNase G-F primer (5'-GATCCTCGAGCTGGCTATGTCGCCAGCG-3') and the RNase G-R primer (5'-GATCGGATCCCGTCTCTCCTTGTCTGCC-3'). The PCR product was digested with XhoI and BamHI and cloned into the same sites in pACYC177.

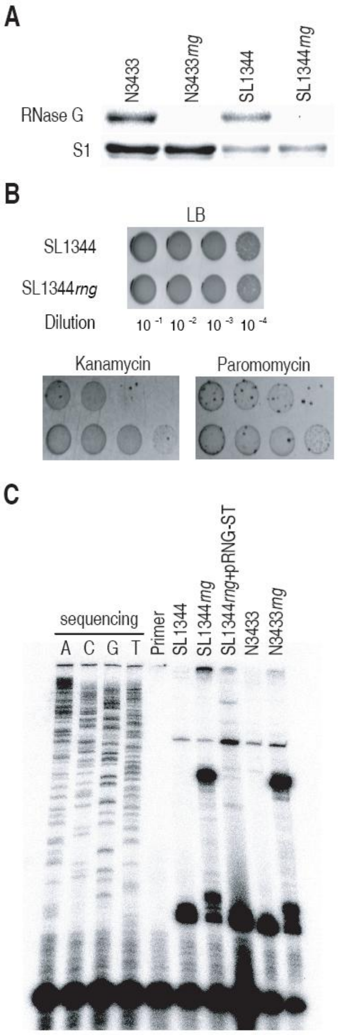

Figure S11. Antimicrobial susceptibilities of MG1655 and MG1655*rng* supplied by Dr. Gloria M. Culver. The wild-type MG1655 and MG1655*rng* strains used by Dr. Gloria M. Culver's group (6) has a different antimicrobial susceptibility compared to those used in the present study. Their wild-type MG1655 and MG1655*rng* strain display a high-level resistance up to 100 µg/ml of kanamycin and 20 µg/ml of chloramphenicol, respectively. Wild-type MG1655 and their *rng* deletion mutant strains used in our study do not exhibit resistance to these antibiotics at the same concentrations. These antimicrobial susceptibility profiles show that Dr. Culver's MG1655 and MG1655*rng* strains do not have the original phenotype of these strains. (1, wild-type MG1655 strain of Dr. Gloria M. Culver ; 2, MG1655*rng* strain of Dr. Gloria M. Culver ; 3, wild-type MG1655 strain used in the present study ; 4, MG1655*rng* strain used in the present study)

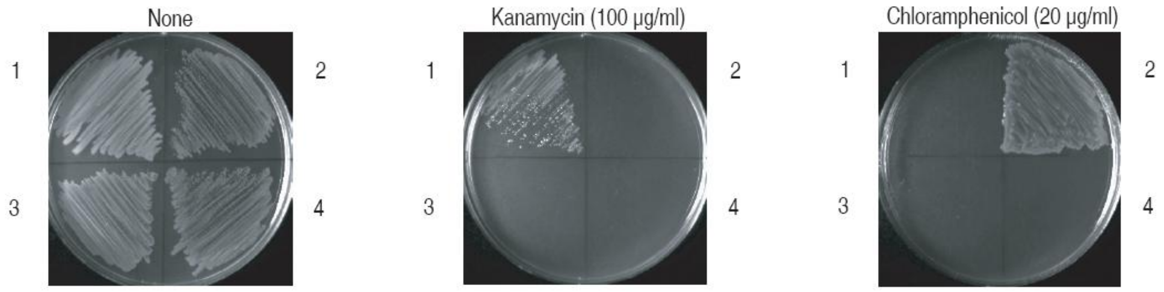

Figure S12. Effects of endoribonuclease deletion on the processing of the 16S rRNA 5' end. For primer extension analysis of the 5' end of 16S rRNA, total RNA, 30S subunits, and 70S ribosomes were purified from strains deleted for each of the endoribonucleases involved in the 5' end processing of 16S rRNA. N3433*rnc* and N3433*rnc-rng* were constructed from N3433 and N3433*rng* by phage P1-mediated transduction using HT115 as a donor strain.

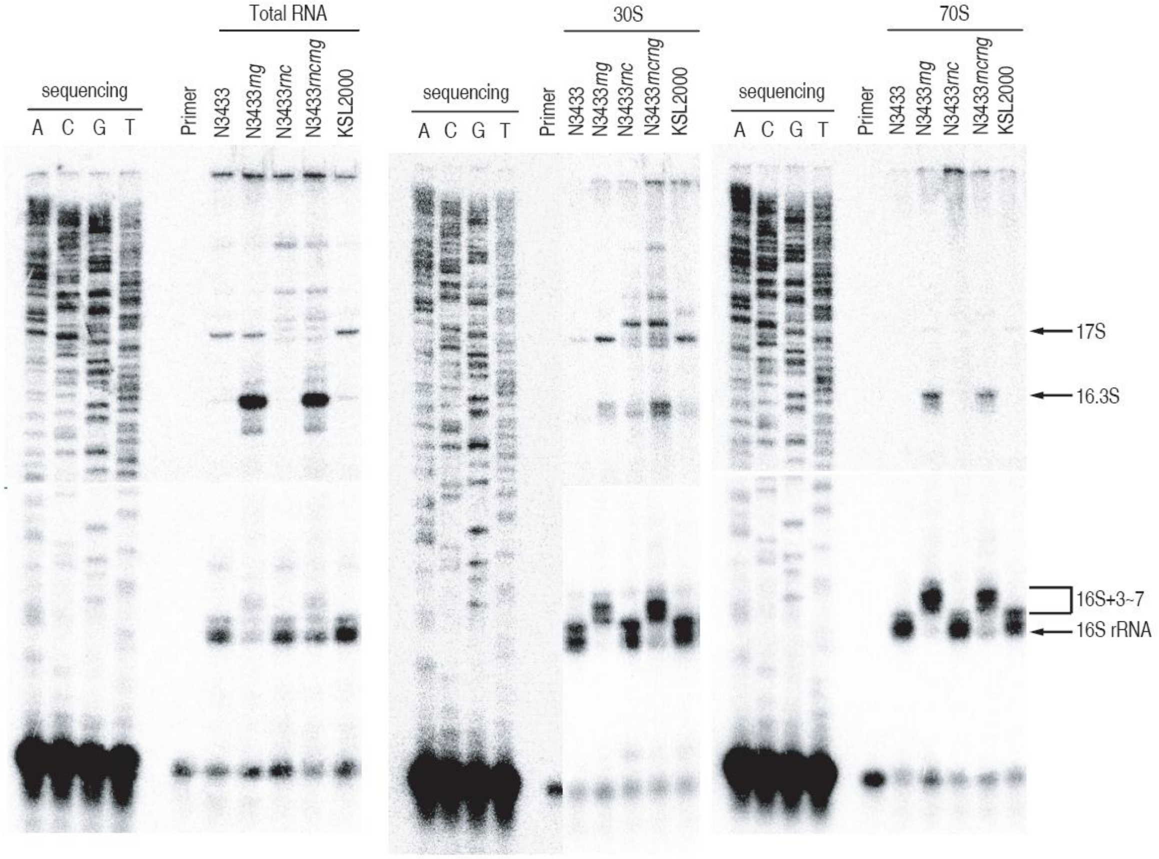

Figure S13. Determination of cellular concentrations of RNase III, RNase E, and RNase G. Three single colonies of strain N3433 were grown overnight in LB media, and diluted by one hundred-fold to cultivate cells to mid-log phase (OD_{600}=0.5~0.6) in the same media. Aliquots of cultures were serially diluted from 10^{-4} to 10^{-6} to determine colony-forming units, and cells were harvested in order to extract proteins. Amounts of N-RNase E, RNase G, and RNase III were analyzed by western blot analysis using the standard proteins purified from KSL2000 cells harboring pNRNE4 (7), BL21(DE3)*rnc105 recA*::Tn9 cells harboring pET15b-rnc (5), and DH5α cells harboring pRNG3 (7), respectively. The His-tagged protein purification was performed as previously described (5).

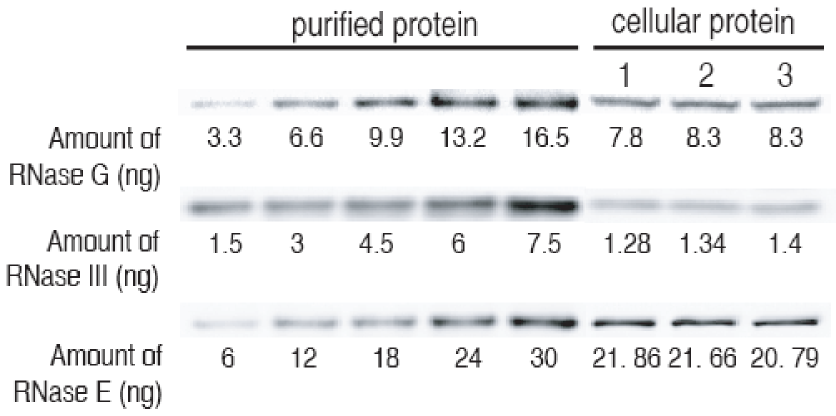

Supplementary References

S1. Gravel, M., Leclerc, D., Melancon, P. and Brakier-Gingras, L. (1989) The conserved 900 stem/loop region in *Escherichia coli* 16S ribosomal RNA is not required for protein synthesis. *Nucleic Acids Res.*, **17**, 2723-2732.

S2. Leclerc, D., Melancon, P. and Brakier-Gingras, L. (1991) Mutations in the 915 region of *Escherichia coli* 16S ribosomal RNA reduce the binding of streptomycin to the ribosome. *Nucleic Acids Res.*, **19**, 3973-3977.

S3. Jerinic, O. and Joseph, S. (2000) Conformational changes in the ribosome induced by translational miscoding agents. *J. Mol. Biol.*, **304**, 707-713.

S4. Sim, S.H., Yeom, J.H., Shin, C., Song, W.S., Shin, E., Kim, H.M., Cha, C.J., Han, S.H., Ha, N.C., Kim, S.W. *et al.* (2010) *Escherichia coli* ribonuclease III activity is downregulated by osmotic stress: consequences for the degradation of *bdm* mRNA in biofilm formation. *Mol. Microbiol.*, **75**, 413-425.

S5. Amarasinghe, A.K., Calin-Jageman, I., Harmouch, A., Sun, W. and Nicholson, A.W. (2001) *Escherichia coli* ribonuclease III: affinity purification of hexahistidine-tagged enzyme and assays for substrate binding and cleavage. *Methods Enzymol.*, **342**, 143-158.

S6. Roy-Chaudhuri, B., Kirthi, N. and Culver, G.M. (2010) Appropriate maturation and folding of 16S rRNA during 30S subunit biogenesis are critical for translational fidelity. *Proc. Natl Acad. Sci. USA*, **107**, 4567-4572.

S7. Lee, K., Bernstein, J.A. and Cohen, S.N. (2002) RNase G complementation of *rne* null mutation identifies functional interrelationships with RNase E in *Escherichia coli*. *Mol. Microbiol.*, **43**, 1445-1456.
